# Climatic and Host-Related Drivers of Gastrointestinal Parasite Dynamics in Domestic Ruminants of North Bengal, India

**DOI:** 10.3390/ani16020338

**Published:** 2026-01-22

**Authors:** Subrata Saha, Manjil Gupta, Rachita Saha, Muhammad Saqib, Elena I. Korotkova, Pradip Kumar Kar

**Affiliations:** 1 Parasitology Laboratory, Department of Zoology, Cooch Behar Panchanan Barma University, Cooch Behar 736101, West Bengal, India; sahabios1989@gmail.com (S.S.); manjilslg@gmail.com (M.G.);; 2 Chemical Engineering Division, School of Earth Sciences and Engineering, National Research Tomsk Polytechnic University, Lenin Avenue 30, 634050 Tomsk, Russia

**Keywords:** gastrointestinal parasites, domestic ruminant, North Bengal, prevalence, seasonality, host-specific drivers, climatic drivers

## Abstract

Gastrointestinal (GI) parasitic infections pose a formidable global challenge to livestock production and continue to affect livestock health and productivity, particularly in tropical and subtropical regions. This study investigated the prevalence, diversity, and epidemiological determinants of GI parasites in 1406 cattle, goats, and sheep from three districts of North Bengal, India (Cooch Behar, Alipurduar, and Jalpaiguri). Parasitological data were analysed using descriptive statistics and inferential methods. Overall prevalence was 69.4%, with cattle showing the highest infection rate (71.62%), followed by sheep (69.30%) and goats (67.19%). Spatial variation was evident among districts, with Cooch Behar recording the highest prevalence (71.20%). Seasonal effects were assessed using Generalized Linear Mixed Models (GLMs), which indicated significantly higher infection probabilities during the monsoon (75.70%) and summer (72.95%) compared with winter (57.78%). The predominant parasite genera identified were *Eimeria* spp., *Strongyloides* spp., and *Fasciola* spp. Host-parasite associations were further explored using Multiple Correspondence Analysis (MCA), revealing distinct clustering patterns, with cattle associated mainly with *Eimeria* spp. and *Strongyloides* spp., goats with *Trichuris* spp. and *Nematodirus* spp., and sheep with *Fasciola* spp. and *Paramphistomum* spp. A species-specific heatmap was used to visualize parasite distribution across host species and seasons, highlighting higher infection intensities during the summer and monsoon periods. Overall, the results demonstrate that GI parasitic infections in North Bengal are influenced by host species and seasonal climatic factors, supporting the implementation of targeted, species- and season-adapted parasite management strategies.

## Simple Summary

This comprehensive study, conducted from November 2020 to December 2024, investigated the prevalence and diversity of gastrointestinal (GI) parasites in 1406 cattle, goats, and sheep across three districts of North Bengal, India. The overall infection rate was 69.4%, with cattle exhibiting the highest prevalence. Infections peaked during the monsoon and summer seasons, particularly in Cooch Behar district, reflecting strong climatic influence. The predominant parasites identified were *Eimeria* spp., *Strongyloides* spp., and *Fasciola* spp. Statistical analyses, including GLM, MCA and species-specific heatmap, revealed distinct host–parasite and seasonal clustering patterns. The findings underscore the significant impact of climatic and ecological factors on GI parasitism and emphasize the need for targeted, season-adapted parasite management strategies to protect livestock health and sustain agricultural productivity.

## 1. Introduction

Gastrointestinal (GI) parasitic infections represent a pervasive and economically devastating impediment to global livestock industries, posing a formidable challenge to food security and animal welfare worldwide [1,2,3]. These infections, instigated by a diverse consortium of helminths and protozoa residing within the digestive tract, profoundly compromise animal health and inflict considerable economic damage through reduced feed intake and conversion efficiency, stunted growth rates, diminished yields of animal products, impaired reproductive performance, escalating veterinary costs, and, in severe instances, outright mortality [4,5]. The magnitude of these losses necessitates a profound epidemiological understanding to formulate efficacious control and prevention strategies [6,7].

The dynamic epidemiological landscape of these parasites is intricately shaped by complex interactions among geographical locale, prevailing climatic conditions, host species and age, and localized animal husbandry practices [8]. Regions characterized by warm, humid climates and extensive grazing systems often provide an optimal ecological niche for the survival, development, and transmission of various parasitic life stages [9]. In this context, climatic drivers such as temperature, humidity, and rainfall are paramount, directly influencing the development rates and survival of free-living parasite stages in the environment [10,11,12].

North Bengal’s humid subtropical climate, marked by high rainfall and extensive wetland systems, provides favourable ecological conditions for the transmission of gastrointestinal parasites. Livestock rearing—primarily cattle, goats, and sheep—is a major livelihood source in the region, making an understanding of parasite distribution particularly relevant for animal health management.

The impact of GI parasites across various ruminant species is well-established. Cattle, as predominant grazers, are susceptible to significant helminthic burdens, including *Fasciola hepatica* and *Haemonchus contortus*, leading to substantial weight loss, reduced milk production, and heightened vulnerability to secondary infections [13,14,15,16]. Goats, often semi-browsers, are particularly prone to severe nematode infections, frequently presenting with acute clinical signs such as debilitating diarrhoea, anaemia, and growth retardation, thereby severely compromising their productivity and survival rates. Similarly, sheep frequently suffer from infections with parasites such as *Eimeria* spp. and *Trichuris* spp., resulting in poor weight gain and elevated mortality, especially in juvenile animals [17,18,19]. Small ruminants, including goats and sheep, also exhibit notable susceptibility to trematode infections such as *Fasciola* spp. and *Paramphistomum* spp. [20].

Despite the critical importance of understanding the prevalence and impact of GI parasites on livestock health and productivity, there remains a notable deficit of comprehensive, region-specific epidemiological data for North Bengal. Previous global studies have consistently highlighted the significant influence of host age, seasonal variations, and management practices on the prevalence of these infections. Therefore, this study was meticulously designed to elucidate the specific diversity and prevalence of gastrointestinal parasitic infections in cattle, goats, and sheep across the districts of Cooch Behar, Alipurduar, and Jalpaiguri.

The insights garnered from this research are poised to inform the development and implementation of effective, localized, species-specific, and season-adapted parasite control strategies [10], ultimately aiming to enhance livestock health, improve productivity, and fortify the agricultural economy within the North Bengal region. This research is critical for developing evidence-based interventions that address the unique climatic and host-related challenges faced by domestic ruminants in this vital agricultural region.

## 2. Materials and Methods

### 2.1. Study Area

**Figure 1:**
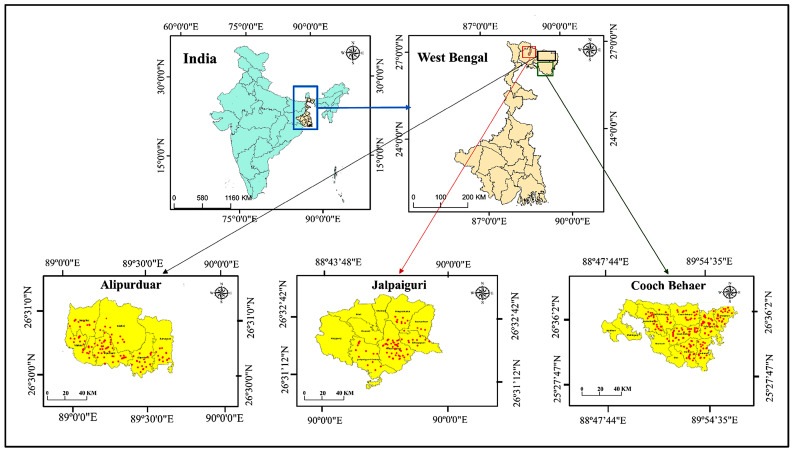
Map of North Bengal, India, showing the three study districts—Cooch Behar, Alipurduar, and Jalpaiguri—in the state of West Bengal. Red points represent the GPS-recorded faecal sample collection sites for cattle, goats, and sheep. This map illustrates the spatial distribution of sampling locations across the study region.

The study was conducted in three districts of North Bengal, India—Cooch Behar, Alipurduar, and Jalpaiguri—each characterised by a humid subtropical climate with high annual rainfall and dense river networks. These environmental conditions favour the development and survival of gastrointestinal parasite eggs and larvae, particularly during the monsoon. Livestock farming involving cattle, goats, and sheep forms a major component of the rural economy in these districts. All faecal sampling sites were georeferenced and mapped to ensure systematic and comprehensive spatial coverage across the region. (Figure 1).

### 2.2. Study Design and Sample Size Determination

A descriptive, cross-sectional study design employing a random sampling method was utilized to select the study animals. The required sample size was calculated using the formula of Thrusfield et al. (2017) [21]:

$$N=\frac{Z^{2}\times P_{exp}(1-P_{exp})}{d^{2}}$$

where

- N = required sample size
- $Z^{2}$ = statistical value for a 95% confidence level (1.96)
- $P_{exp}$ = expected prevalence (assumed to be 50%)
- d = desired absolute precision (0.05)

Because no prior prevalence estimate was available for the study area, an expected prevalence of 0.05 was used, as this value yields the maximum required sample size and therefore ensures adequate statistical power under worst-case uncertainty. Based on this formula, the required sample size was calculated as 384 animals. To enhance the robustness and representativeness of the study, a total of 1406 animals were ultimately samples, comprising 518 cattle, 445 goats and sheep.

### 2.3. Faecal Sample Collection

A total of 1406 freshly voided faecal samples (approximately 50 g each) were collected from cattle, goats, and sheep during November 2020 to December 2024. Animals were primarily managed under traditional extensive conditions in the rural areas within the three designated study districts. Samples were collected early in the morning, immediately following defecation from the ground using sterile plastic gloves to prevent contamination. Each sample was placed into a sterile plastic container with a secure lid and assigned a unique alphanumeric code that linked it to the hostspecies, collection site, GPS coordinates, date, and season [22,23,24]. To prevent further embryonation or degradation of parasitic stages, 10% formalin was added to each collected sample [25]. The preserved samples were then transported under cold chain conditions (in insulated boxes with ice packs) to the laboratory for subsequent parasitological examination. Samples were stored at 4 °C until analysis [24,25,26].

### 2.4. Coprological Analysis

For the comprehensive epidemiological investigation of gastrointestinal parasite infection, various coprological analyses were performed on the collected faecal samples. The standard coprological methods typically involve techniques such as flotation and sedimentation to detect helminth eggs and protozoan oocysts. Parasite eggs and oocysts were identified based on their characteristic morphological appearance and size under a light optical microscope [13,19,26,27,28,29,30].

#### 2.4.1. Modified Saturated Saline Flotation Method

This technique was employed for the detection of lighter helminth eggs (e.g., nematode and cestode eggs) and coccidian oocysts. Briefly, approximately 4 g of faecal material from each sample were thoroughly mixed with 60 mL of saturated saline solution. The mixture was then filtered through a sieve (e.g., 250 µm pore size) to remove large debris. The filtrate was transferred to a centrifuge tube and centrifuged at 800× *g* for 5 min. A coverslip was carefully placed on the surface of the supernatant, allowed to stand for 3 min, and then transferred to a clean glass slide for microscopic examination [27,28,31].

#### 2.4.2. Modified Ritchie’s Formol-Ether Sedimentation Technique

This method was utilized to concentrate heavier parasite eggs (e.g., trematode eggs) and protozoan ocysts. Approximately 4 g of faecal material were mixed with 60 mL of tap water in a disposable plastic cup. This suspension was poured into an Erlenmeyer flask, and additional water was added to achieve a total volume of 100 mL. A 15 mL aliquot of this mixture was transferred to a graduated conical centrifuge tube and centrifuged at 500× *g* for 2 min. The supernatant was discarded, and the sediment was resuspended in 12 mL of water. Subsequently, 3 mL of diethyl ether was added, and the homogenate was centrifuged again at 300× *g* for 2 min. The resulting supernatant and debris layer were removed, and the remaining sediment was diluted with a few drops of distilled water. A 20 µL aliquot of this diluted sediment was placed onto a clean glass slide, covered with a coverslip, and examined microscopically [19,30,32,33].

### 2.5. Parasite Identification

Parasite eggs and oocysts were identified based on their characteristic morphological features, including size, shape, color, and internal structures, using a light optical microscope (Axioscope 5, Carl Zeiss, Bengaluru, India) at various magnifications (e.g., 100×, 400× for detailed examination; 10×, 40× objective lenses for initial screening. All identifications were performed according to established standard keys and reference atlases [27,33,34,35]. The gastrointestinal parasites identified in this study were classified into the following major taxonomic categories: nematodes, cestodes, trematodes, and protozoa.

### 2.6. Data Management and Quality Assurance

All data generated during field sampling, laboratory examination, and statistical analysis were managed using a standardized datasheet-based framework to ensure data integrity, analytical transparency, and reproducibility. Each faecal sample was assigned a unique alphanumeric identifier linking host species (cattle, goat, or sheep), sampling location (district and GPS coordinates), date of collection, and season [36]. This identifier was retained consistently across all datasets and analytical stages.

Raw parasitological observations were entered into structured electronic datasheets prepared in Microsoft Excel. For statistical analysis, infection status for each gastrointestinal parasite taxon was encoded as a binary (binomial) variable, with presence recorded as “1” and absence as “0”. This binomial coding scheme was applied uniformly to all identified parasite taxa, including *Eimeria* spp., *Fasciola* spp., *Paramphistomum* spp., *Moniezia* spp., *Strongyloides* spp., *Trichostrongylus* spp., *Nematodirus* spp., *Trichuris* spp., and *Oxyuris* spp., as well as to overall gastrointestinal parasite infection status. The use of binomial response variables enabled consistent modeling of parasite occurrence across hosts, seasons, and geographic areas [37,38].

Data quality assurance procedures were implemented at multiple stages. Initial data entry was verified through independent double-entry checks, and logical consistency among host, spatial, and temporal variables was systematically validated. Parasite identification results were independently confirmed by two experienced parasitologists; samples yielding uncertain or conflicting diagnoses were re-examined under blinded conditions to minimize observer bias. Prior to inferential analysis, the finalized datasheets were screened for missing values, outliers, and duplicate records. Entries with incomplete metadata were excluded from model-based analyses but retained for descriptive reporting where appropriate [39,40].

All statistical analyses, including Chi-square tests, generalized linear models with binomial error structure, multiple correspondence analysis, and species-specific heatmap visualizations, were conducted using the validated binomial datasets derived directly from the finalized datasheets. Analytical scripts, intermediate datasets, and graphical outputs were systematically archived to ensure full traceability of results and reproducibility of the analytical workflow. This comprehensive data management and quality assurance protocol ensured that all statistical inferences were robust, internally consistent, and directly grounded in the original parasitological observations [39,41,42,43].

### 2.7. Statistical Analysis

All statistical analyses were performed to investigate climatic and host-related drivers of gastrointestinal parasite dynamics in domestic ruminants of North Bengal, India. Data organization and preliminary descriptive statistics, including prevalence (%) and frequency distributions for each parasite taxon, were conducted using Microsoft Excel 2010 [38].

The effects of host species, season, and geographic area on parasite occurrence were evaluated using generalized linear model (GLM) implemented in jamovi statistical software (version 5.0; Sydney, Australia). Parasite infection status was analyzed as a binary response variable (presence/absence) under a binomial error distribution with a logit link function. Separate GLMs were fitted for each of the nine gastrointestinal parasite taxa (*Eimeria* spp., *Fasciola* spp., *Paramphistomum* spp., *Moniezia* spp., *Strongyloides* spp., *Trichostrongylus* spp., *Nematodirus* spp., *Trichuris* spp., and *Oxyuris* spp.), as well as for overall infection status. Host species, season, and geographic area were included as fixed effects, and interaction terms were incorporated to assess combined influences of these factors. Model significance was assessed using analysis of variance (ANOVA), with F statistics and associated *p*-values used to evaluate the contribution of explanatory variables. Model performance was evaluated using R^2^ and adjusted R^2^ values [44].

Multivariate relationships among host species, seasonal patterns, geographic distribution, and parasite taxa were explored using Multiple Correspondence Analysis (MCA). MCA was conducted in Python (version 3.10; Python Software Foundation, Wilmington, DE, USA) using an indicator matrix derived from one-hot encoding of categorical variables. Singular value decomposition was applied to extract principal dimensions, and the first two dimensions were retained for interpretation based on inertia values. MCA biplots were generated to visualize clustering patterns, category associations, and co-occurrence structures among parasite taxa [45].

Additionally, heatmap analysis was performed in Python to visualize parasite prevalence across host species and seasons. Prevalence values were standardized and displayed using a sequential color gradient to highlight variation in infection intensity and seasonal trends. Data processing and visualization were carried out using the pandas (version 2.3.3; New York, NY, USA), numpy (version 2.2; NumPy Developers, Cheyenne, WY, USA), matplotlib (version 3.10.8), and seaborn libraries (version 0.13.2) [46].

## 3. Results

The comprehensive epidemiological investigation into gastrointestinal (GI) parasite dynamics in domestic ruminants across North Bengal, India, yielded a dataset that revealed patterns of infection influenced by host species, geographic location, and pronounced seasonal climatic variations. This section presents a detailed analysis of the prevalence, distribution, and variations of these parasitic infections, integrating findings from descriptive statistics, Chi-square analyses, Generalized Linear Models (GLMs) [47,48], Multiple Correspondence Analysis (MCA) [49,50], and species-specific heatmaps [51,52]. The results collectively establish a quantitative ecological framework for understanding parasite transmission in this humid subtropical region.

### 3.1. Overall Prevalence and Distribution Patterns

A total of 1406 faecal samples were randomly collected from domestic ruminants (cattle, goats, and sheep) across the three districts of North Bengal: Cooch Behar, Alipurduar, and Jalpaiguri. Of these, 977 samples, representing a substantial overall prevalence of 69.4%, tested positive for one or more GI parasites. This high prevalence underscores the pervasive nature of these infections within the region’s livestock populations. The distribution of these infections was not uniform but showed significant variation across host species, geographic areas, and seasons, indicating complex underlying epidemiological drivers.

#### 3.1.1. Host-Specific Prevalence

Among the three domestic ruminant species examined, cattle demonstrated the highest overall susceptibility to GI parasitic infections. Out of 518 cattle samples, 373 were positive, resulting in an infection rate of 72.01% (Table 1). This elevated prevalence in cattle is likely attributable to their predominant grazing behaviour, which increases their exposure to contaminated pastures where infective larval stages and oocysts are abundant. Sheep followed with a considerable prevalence of 69.30% (Table 1). Goats recorded a slightly lower, yet still substantial, prevalence of 66.74% (Table 1). The observed differences in host-specific prevalence suggest varying degrees of susceptibility, exposure patterns linked to feeding habits, or potentially differences in immune responses among the ruminant species.

#### 3.1.2. Geographical Prevalence

**Figure 2:**
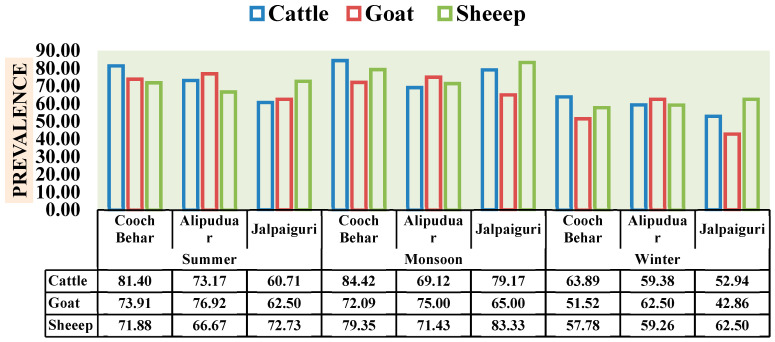
Seasonal prevalence of gastrointestinal parasites in cattle, goat and sheep across three districts of North Bengal (Cooch Behar, Alipurduar and Jalpaiguri).

Geographical location played a significant role in shaping the observed infection rates. Cooch Behar district recorded the highest overall prevalence at 70.80% (536 positive samples out of 757), indicating a particularly high burden in this area (Table 2). Alipurduar followed with a prevalence of 68.71% (325 positive samples out of 473), while Jalpaiguri exhibited the lowest prevalence at 65.90% (116 positive samples out of 176) (Table 2). This spatial gradient in prevalence is visually represented in Figure 2, which compares infection rates across the three districts for cattle, goats, and sheep. For instance, cattle in Cooch Behar showed a particularly high prevalence of 77.02%, whereas goats in Jalpaiguri had the lowest prevalence at 56.92% (Table 1). This geographical heterogeneity likely reflects differences in microclimatic conditions, grazing intensities, and the proximity to wetlands, all of which critically influence the transmission ecology of various helminths and protozoans. The low-lying, flood-prone areas of Cooch Behar and parts of Alipurduar, for example, provide ideal conditions for the survival and proliferation of intermediate hosts for trematodes.

#### 3.1.3. Seasonal Prevalence

A pronounced seasonal pattern emerged as a critical determinant of infection dynamics, directly correlating with favourable environmental conditions. The monsoon season exhibited the highest overall prevalence rate at 75.70% (352 positive samples out of 465), followed closely by the summer season with 72.95% (391 positive samples out of 536) (Table 2). In stark contrast, the winter season recorded a significantly lower prevalence of 57.78% (234 positive samples out of 405) (Table 2). This striking seasonal variation, with a Chi-square value of 37.68 and a *p*-value of <0.001 (Table 2), underscores the paramount role of rainfall and ambient temperature in facilitating the survival, development, and transmission of infective larval stages and oocysts.

A comparative analysis of parasite prevalence across host species (cattle, goat, and sheep) and seasons (summer, monsoon, and winter) revealed pronounced spatial and temporal variations (Figure 2). Among all hosts, cattle consistently exhibited the highest prevalence, reaching 84.42% in Cooch Behar during the monsoon, followed by 81.40% in summer, while the lowest infection rate (63.89%) was observed in winter. Goats showed moderate infection levels, ranging from 76.92% (Alipurduar, summer) to 42.86% (Jalpaiguri, winter), whereas sheep demonstrated more fluctuating patterns, peaking at 83.33% (Jalpaiguri, monsoon) and declining to 57.78% (Cooch Behar, winter).

#### 3.1.4. Parasite Taxa Prevalence

**Figure 3:**
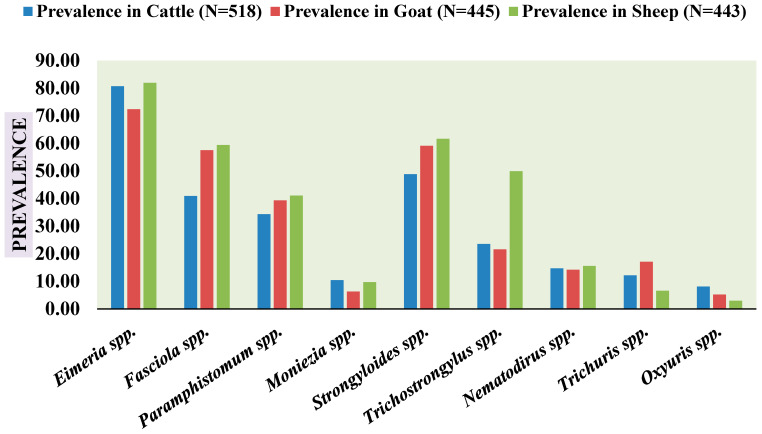
Parasite species-specific prevalence in domestic herbivores (cattle, goat and sheep) in three districts of North Bengal (Cooch Behar, Alipurduar and Jalpaiguri).

Regarding the specific parasite taxa identified, *Eimeria* spp. were the most frequently detected, accounting for 29.73% of all infections (418 positive samples out of 1406) (Table 2). This dominance highlights the widespread presence and significant impact of coccidian infections in the region. *Strongyloides* spp. followed with a prevalence of 17.99% (253 samples), indicating a substantial burden from this nematode (Table 2). Trematode infections were also prominent, with *Fasciola* spp. at 15.08% (212 samples) and *Paramphistomum* spp. at 12.66% (178 samples) (Table 2). Other identified genera, including *Trichostrongylus* spp. (8.68%), *Nematodirus* spp. (5.41%), *Trichuris* spp. (4.48%), *Moniezia* spp. (3.84%), and *Oxyuris* spp. (2.99%), were present at lower frequencies (Table 2). The species-specific prevalence across domestic herbivores is graphically presented in Figure 3, clearly illustrating the relative abundance of each parasite type and confirming the dominance of *Eimeria* spp. and *Strongyloides* spp. The lower prevalence of *Oxyuris* spp., *Nematodirus* spp., and *Moniezia* spp. (all below 6%) suggests either limited environmental resilience of their infective stages or host-specific transmission barriers under the prevailing regional climatic conditions (Table 2).

### 3.2. Host, Spatial, and Seasonal Associations (Chi-Square Analysis)

The contingency-based χ^2^ analyses demonstrated strong non-random associations between infection status and all categorical factors (host, area, season, and parasite type), each showing *p* < 0.001. Host species contributed most strongly to the variance structure, explaining approximately 21.8% of total deviance, followed by area (19.3%) and season (15.7%). Notably, *Eimeria* spp. (29.73%) and *Strongyloides* spp. (17.99%) displayed the highest parasite-specific χ^2^ values, confirming their epidemiological dominance and persistence across host and environmental strata.

Spatial heterogeneity was more pronounced in trematode infections (*Fasciola* and *Paramphistomum* spp.), which were concentrated in low-lying, flood-prone pastures of Cooch Behar and southern Alipurduar. Conversely, nematode and cestode infections showed more uniform distribution across districts, suggesting less dependence on intermediate host ecology (Table 2).

### 3.3. Generalized Linear Model (GLM) Analysis

Generalized linear modelling (binomial framework; reported here in the OLS/ANOVA presentation given in the Supplementary GLM file) revealed parasite-specific responses to host, season and geographic area. For transparency we report model fit (R^2^/adjusted R^2^), omnibus ANOVA outcomes and the most important interactions for each taxon; full coefficient tables are provided in the Supplementary Tables.

**Figure 4:**
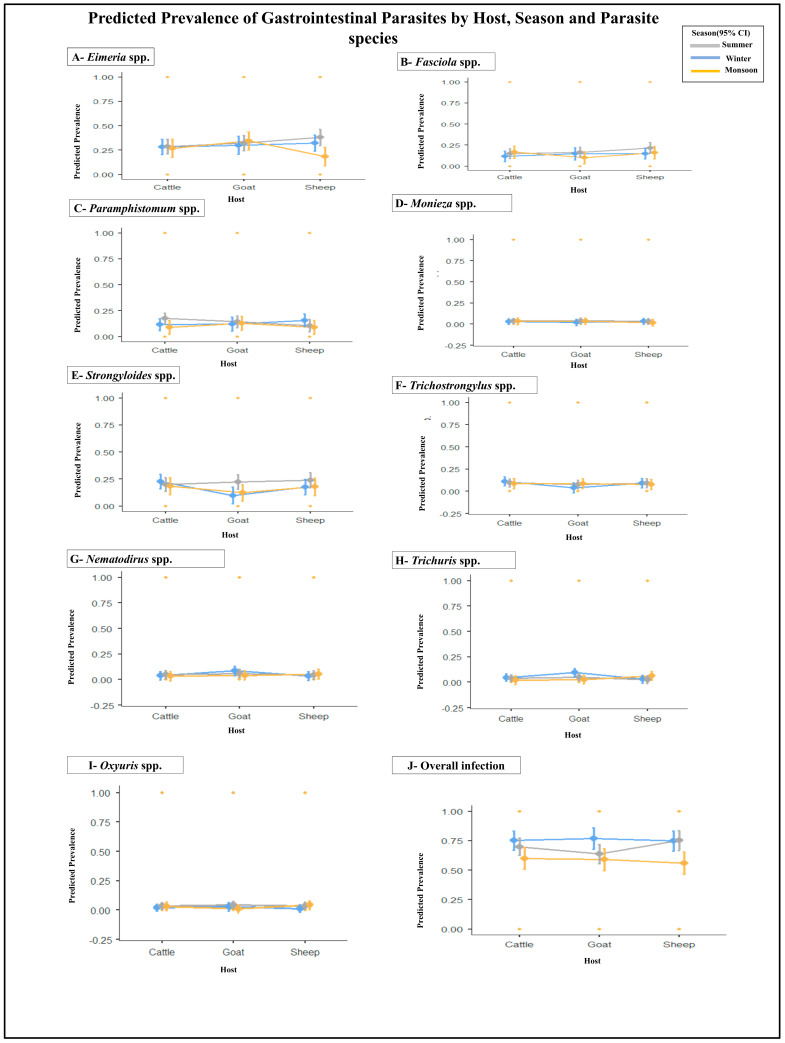
Predicted prevalence of gastrointestinal parasites in domestic ruminants based on GLM analysis. Each panel shows modeled prevalence across host species (Cattle, Goat, Sheep) and seasons (Summer, Monsoon, Winter) for individual parasite taxa: (**A**) *Eimeria* spp.; (**B**) *Fasciola* spp.; (**C**) *Paramphistomum* spp.; (**D**) *Moniezia* spp.; (**E**) *Strongyloides* spp.; (**F**) *Trichostrongylus* spp.; (**G**) *Nematodirus* spp.; (**H**) *Trichuris* spp.; (**I**) *Oxyuris* spp.; and (**J**) overall infection. Points represent predicted means with 95% confidence intervals. Higher prevalence was observed during the Monsoon season, with marked host-specific variations.

Overall infection (presence of one or more parasite taxa) was explained modestly by the full model (R^2^ = 0.0422, adj. R^2^ = 0.0242). The omnibus ANOVA for the overall model was highly significant (Model SS = 12.5877, df = 26, F = 2.338, *p* < 0.001), and season was the strongest single predictor (SS = 4.5392, F = 10.962, *p* < 0.001), indicating clear seasonal structuring of overall parasite risk across districts and hosts. Area and Host main effects were not significant at the omnibus level, although several interaction terms showed local significance (see Supplementary Table S10 and Figure 4J).

Taxon-level summaries follow (statistics quoted as model R^2^/adj. R^2^ and omnibus *p*-values; notable interactions or significant terms are described where present).

*Eimeria* spp.—The model explained a small proportion of variance (R^2^ = 0.02644; adj. R^2^ = 0.00808). The omnibus ANOVA did not reach conventional significance (Model: F (26,1379) = 1.440, *p* = 0.071), and main effects of Host, Season and Area were non-significant. A marginal Season × Area effect approached significance (*p* ≈ 0.055), suggesting weak spatial–seasonal structuring for *Eimeria* oocyst detection. Fixed-effect parameter estimates were small and non-significant for primary contrasts (e.g., Goat vs. Cattle; Sheep vs. Cattle) (Supplementary Table S1 and Figure 4A).

*Fasciola* spp.—The model showed low explanatory power (R^2^ ≈ 0.0182, adj. ≈ 0.000), with a non-significant omnibus test (*p* = 0.485). However, the Host × Area interaction approached the significance boundary (Host × Area: F ≈ 2.382, *p* = 0.050), consistent with spatial heterogeneity in *Fasciola* risk linked to local environmental suitability for intermediate hosts. Individual host contrasts were generally non-significant (Supplementary Table S2 and Figure 4B).

*Paramphistomum* spp.—The model performed comparatively better (R^2^ = 0.0324; adj. R^2^ = 0.0141) and the omnibus test was significant (Model: F = 1.774, *p* = 0.010). Notably, the Host × Area interaction was statistically significant (F ≈ 4.557, *p* = 0.001), indicating that the effect of host species on *Paramphistomum* presence differs among districts—a pattern consistent with local grazing or habitat differences that affect trematode transmission. Main effects for Host and Season were weak or non-significant in isolation (Supplementary Table S3 and Figure 4C).

*Moniezia* spp.—Low model fit (R^2^ ≈ 0.0170) and non-significant model omnibus (*p* = 0.581). Area showed some contribution (Area SS and F values larger than other main effects), but overall *Moniezia* prevalence did not show pronounced seasonality in the GLM framework (Supplementary Table S4 and Figure 4D).

*Strongyloides* spp.—The model explained little variance (R^2^ ≈ 0.0196; adj. ≈ 0.001). The omnibus test did not reach significance (*p* ≈ 0.386), although Season showed a trend (*p* ≈ 0.086) and certain Host × Season × Area contrasts were significant in pairwise parameter tests, suggesting localized seasonal peaks for some host–area combinations (Supplementary Table S5 and Figure 4E).

*Trichostrongylus* spp.—Low explanatory power (R^2^ = 0.0187; adj. ≈ 0.00025) and a non-significant omnibus test (*p* = 0.445). Most main effects and interactions were non-significant, though selected Host × Area contrasts had elevated F/t values consistent with moderate spatial heterogeneity (Supplementary Table S6 and Figure 4F).

*Nematodirus* spp.—Modest fit (R^2^ = 0.02088; adj. R^2^ = 0.00242); the omnibus model *p* = 0.296. A higher-order Host × Season × Area interaction reached significance (*p* = 0.032), indicating that *Nematodirus* prevalence is structured by a three-way combination of host, season and location (i.e., certain host groups show seasonal peaks in some districts). Main effects alone were weak (Supplementary Table S7 and Figure 4G).

*Trichuris* spp.—Low explained variance (R^2^ ≈ 0.0239) and non-significant omnibus test (*p* = 0.143). Season × Area showed evidence of contribution (Season × Area: F ≈ 2.996, *p* = 0.018), suggesting spatial differences in seasonal exposure that affect *Trichuris* egg survival and detection (Supplementary Table S8 and Figure 4H).

*Oxyuris* spp.—The weakest model overall (R^2^ ≈ 0.0183; adj. R^2^ ≈ 0), omnibus *p* = 0.480. No consistent host or season main effects were detected; *Oxyuris* presence appears sporadic and highly localized (Supplementary Table S9 and Figure 4I).

### 3.4. Multivariate Patterns from Multiple Correspondence Analysis (MCA)

Multiple Correspondence Analysis was conducted to examine the multivariate associations among host species, season, and geographic area in relation to parasite distribution. The first two MCA dimensions accounted for the major structural variation in the categorical dataset, with Dimension 1 explaining 2.06% of the inertia and Dimension 2 explaining 21.34%. Although the total inertia is modest, the patterns captured by these axes reflect meaningful ecological and management-related gradients.

#### 3.4.1. Individual-Level Patterns

**Figure 5:**
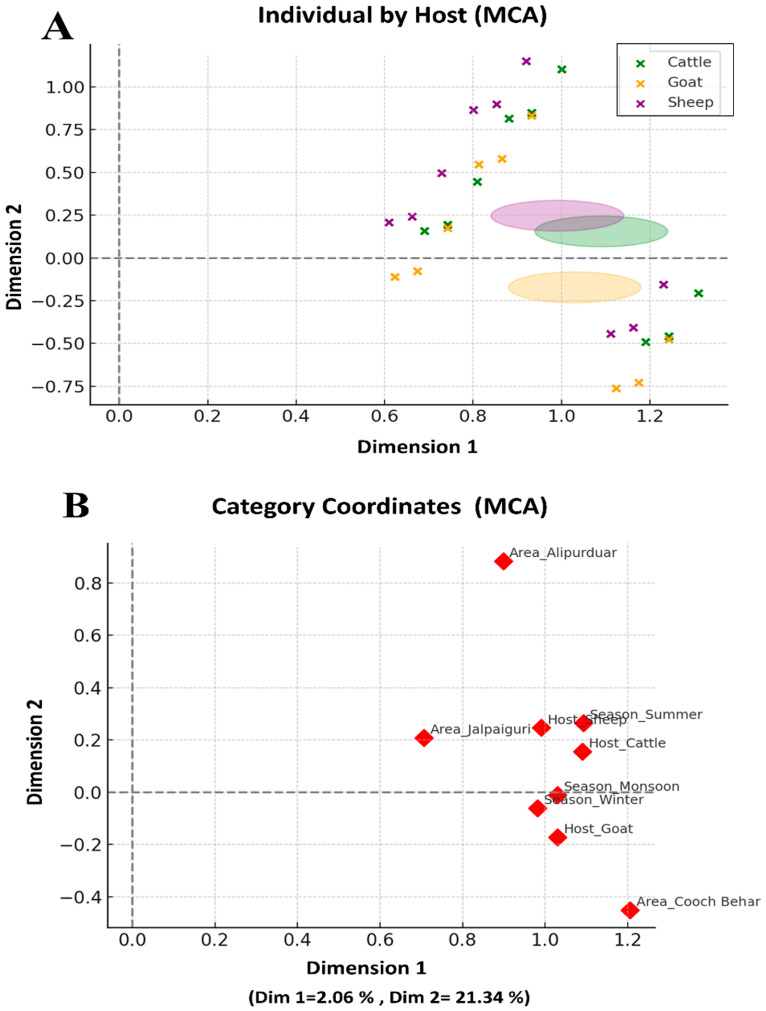
Multiple Correspondence Analysis (MCA) of Host, Seasonal, and Geographic Factors: This figure shows the MCA performed on categorical variables (host species, season, area) to explore multivariate patterns associated with gastrointestinal parasite occurrence. MCA was applied to a one-hot encoded indicator matrix, and the first two dimensions (Dim 1 = 2.06%; Dim 2 = 21.34% inertia) represent the main structural gradients in the dataset. (**A**) Projection of individual animals, colored by host species. Elliptical envelopes indicate the dispersion of each host group. Sheep tend to align positively along Dimension 2, suggesting a stronger association with seasonal or management factors, while goats are more widely dispersed and cattle cluster near the origin. (**B**) Centroid positions of all categorical levels. Alipurduar shows a distinct positive association with Dimension 2, whereas Cooch Behar loads negatively, and Jalpaiguri remains near the origin. Summer aligns positively with Dimension 2, while Winter and Monsoon cluster more centrally. Host centroids show moderate separation, with Sheep again positively associated with Dimension 2. Together, these patterns illustrate how host identity and environmental context jointly structure parasite-related ecological variation.

Projection of individual animals (Figure 5A) demonstrated partial but interpretable clustering by host species. Sheep showed a clear tendency to align positively along Dimension 2, indicating a stronger association with the categorical factors driving variation on this axis. Goats displayed moderate dispersion across both axes, suggesting a wider range of exposure conditions or management variability. Cattle clustered near the origin, reflecting a more generalist or mixed ecological profile with a weaker association to the structural gradients represented in the MCA. Elliptical envelopes further illustrated that sheep formed the most distinct grouping, while cattle and goats exhibited greater overlap.

#### 3.4.2. Category-Level Patterns

Category coordinates (Figure 5B) revealed consistent patterns across host, season, and area. Among geographic regions, Alipurduar loaded strongly and positively on Dimension 2, suggesting distinct environmental or management characteristics relative to the other districts. Cooch Behar showed negative alignment on the same axis, while Jalpaiguri occupied a central position close to the origin, indicating intermediate or heterogeneous conditions. Seasonal categories also separated along Dimension 2: Summer aligned positively, whereas Winter and Monsoon remained near the axis origin, reflecting limited differentiation between these cooler or wetter periods. Host centroids mirrored individual-level patterns, with Sheep positively associated with Dimension 2, and Goat and Cattle situated closer to the central region.

#### 3.4.3. Ecological Interpretation

Overall, the MCA results indicate that Dimension 2 captures the principal ecological gradient combining host-related factors with seasonal and geographic influences, while Dimension 1 represents minor spatial contrasts. The distinct positioning of sheep suggests that their grazing behavior, management practices, or susceptibility may interact more strongly with environmental conditions that shape parasite exposure. The contrasting positions of Alipurduar and Cooch Behar further support the influence of regional environmental variability on parasite-related categorical structure. These multivariate patterns highlight the interconnected effects of host identity, climate, and geography on parasite epidemiology in ruminant populations.

### 3.5. Parasite Species-Specific Prevalence Patterns (Heatmap Analysis)

The parasite prevalence heatmap revealed clear differences among host species and seasons in North Bengal. Among all taxa examined, *Eimeria* spp. consistently showed the highest prevalence across cattle, goats, and sheep, with peak values during the summer and monsoon seasons. *Strongyloides* spp. and *Fasciola* spp. also demonstrated elevated infection levels, particularly in goats and sheep during summer, indicating that warm and humid environmental conditions strongly favour their transmission. In contrast, *Moniezia* spp., *Trichuris* spp., and *Oxyuris* spp. exhibited comparatively lower prevalence across all host–season combinations, with only minor seasonal fluctuations. Seasonal differences were clearly reflected in the heatmap: most parasite groups showed increased prevalence during monsoon and summer, whereas winter values remained uniformly lower, highlighting the role of temperature, moisture, and pasture contamination in shaping parasite dynamics. Host-level patterns were also evident; cattle and sheep typically displayed higher prevalence for several parasite taxa, while goats showed more variable but seasonally responsive infection profiles.

**Figure 6:**
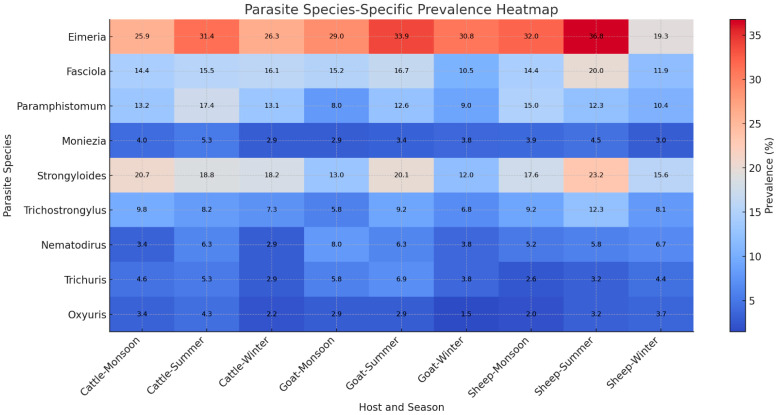
Species-specific prevalence (%) of gastrointestinal parasites across host and seasonal combinations in ruminants of North Bengal, India. The heatmap illustrates infection intensity, with darker shades indicating higher prevalence. *Eimeria* spp., *Strongyloides* spp., and *Fasciola* spp. were most prevalent during the summer and monsoon seasons, particularly in cattle and goat.

Overall, the heatmap effectively summarizes how parasite species differ in their ecological responses, illustrating strong seasonal peaks, host-associated susceptibility, and notable co-occurrence trends among common gastrointestinal parasites. These patterns emphasize the combined influence of environmental conditions, grazing behavior, and host biology in determining parasite burdens across North Bengal (Figure 6).

### 3.6. Integrated Interpretation of Epidemiological Drivers

The combined outcomes of the univariate, GLM, MCA, and heatmap analyses indicate that gastrointestinal parasite prevalence in North Bengal is governed by host-specific biology, climatic seasonality, and spatial hydro-ecological factors. These drivers collectively regulate parasite transmission by influencing exposure patterns, larval development, and the persistence of infective stages in the environment.

The GLM and MCA results showed that infection dynamics are not random but structured by predictable ecological interactions. High χ^2^ and GLM coefficients for *Eimeria*, *Fasciola*, and *Strongyloides* highlight their sensitivity to climatic variation, positioning them as sentinel species for environmental change. The MCA ordination revealed distinct clustering of host–parasite relationships, with *Eimeria* and *Strongyloides* infections dominating in cattle, *Trichuris* and *Nematodirus* in goats, and *Fasciola* and *Paramphistomum* in sheep.

Heatmap analysis further visualized species-specific infection intensity, showing that *Eimeria* spp., *Strongyloides* spp., and *Fasciola* spp. were most prevalent during the summer and monsoon seasons, particularly in cattle and goats. These patterns underscore the strong influence of humidity and temperature on parasite proliferation. Overall, the integration of GLM, MCA, and heatmap findings demonstrates that parasite transmission in North Bengal is shaped by the combined effects of host ecology and seasonal climate, providing a scientific basis for designing targeted, seasonally adaptive parasite control programs in ruminants.

## 4. Discussion

### 4.1. Summary of Study Aims and Key Results

This study quantified the diversity, prevalence and seasonal dynamics of gastrointestinal (GI) parasites infecting domestic ruminants (cattle, goats and sheep) across three districts of North Bengal, India (Cooch Behar, Alipurduar and Jalpaiguri) between November 2020 and December 2024. A total of 1406 faecal samples were examined using standard coprological methods (modified saturated saline flotation, Ritchie’s formol–ether sedimentation and modified McMaster counting). The overall prevalence of GI parasites was 69.4% (977/1406). Host-specific prevalences were 72.01% in cattle (373/518), 66.39% in goats (297/445) and 69.30% in sheep (307/443). Spatially, Cooch Behar recorded the highest district-level prevalence (e.g., cattle 77.02% in Cooch Behar). Seasonally, infections peaked in the monsoon and summer periods. The dominant parasite genera identified were *Eimeria* spp., *Strongyloides* spp. and *Fasciola* spp. Multivariate approaches (Generalized Linear Models and Multiple Correspondence Analysis), together with a species-specific heatmap, revealed distinct host–parasite–season clustering: cattle associated with *Eimeria* and *Strongyloides*; goats with *Trichuris* and *Nematodirus*; and sheep with Fasciola and *Paramphistomum.* These analyses demonstrated predictable ecological structuring of infection patterns in this humid subtropical system

### 4.2. Interpretation of Results

The high overall prevalence (~69.4%) indicates pervasive GI parasitism among domestic ruminants in North Bengal. The slightly higher prevalence observed in cattle compared to small ruminants likely reflects differences in feeding behaviour and exposure: cattle are predominately grazers and thus experience higher contact with faecally contaminated pastures, which is consistent with their clustering with *Eimeria* and *Strongyloides* in GLM and MCA outputs. Seasonal peaks in the monsoon and summer align with the region’s humid subtropical climate—elevated temperature and rainfall create favourable conditions for egg embryonation, larval development and survival of infective stages, explaining increased transmission during wet months [12,53]. The predominance of *Fasciola* and *Paramphistomum* in sheep points to hydro-ecological drivers (presence of snail intermediate hosts and marshy grazing areas) consistent with the observed spatial heterogeneity among districts [20]. Host-specific parasite assemblages likely reflect combined effects of grazing behaviour, differential susceptibility and management practices (e.g., housing, anthelmintic use), while frequent mixed infections suggest co-occurrence that may influence intensity and clinical impacts [38,54]. Overall, GLM and MCA results indicate infection dynamics structured by host species, season and parasite taxa, with sampling area contributing spatial heterogeneity captured by the random-effects structure.

### 4.3. Ecological and Epidemiological Implications

The elevated and seasonally variable burden of GI parasites has direct implications for animal health and productivity in the region. High monsoon and summer prevalence suggests parasite control programmes should be seasonally targeted to coincide with peak transmission windows [42,55]. Host-specific assemblages imply that interventions must be tailored by species—targeting coccidia and nematodes in cattle, nematode control in goats, and trematode (fluke) control in sheep [54,56,57]. Hydro-ecological influences on fluke transmission underscore the value of integrating landscape-level risk assessment (e.g., mapping snail habitats and wet grazing areas) into management plans [9,58,59]. Frequent mixed infections increase the likelihood of subclinical production losses and complicate diagnosis and treatment. Persistent high prevalence also raises concern for potential anthelmintic selection pressure if treatments are unmonitored or poorly timed [60]. The spatial heterogeneity across districts supports localized surveillance and geographically targeted interventions rather than uniform strategies across the region.

### 4.4. Study Limitations

Several limitations warrant consideration: (1) Diagnostic resolution—coprological methods provided genus-level morphological identifications but cannot reliably resolve closely related species or detect prepatent infections; molecular confirmation was not included. (2) Representativeness—although the sample size (*n* = 1406) and random sampling across seasons and districts improve robustness, study animals were primarily reared under semi intensive systems and findings may not fully represent purely extensive pastoral or entirely stall-fed populations. (3) Management and treatment data—detailed, standardized information on prior anthelmintic treatments, nutritional status and husbandry practices were not incorporated into models and could confound associations. (4) Clinical and production linkage—the study quantified prevalence and infection intensity (EPG/OPG) but did not directly link parasitological findings to production metrics (e.g., weight gain, milk yield) or clinical outcomes. (5) Environmental and intermediate-host data—snail surveys and direct environmental measures relevant to trematode transmission were not performed, limiting mechanistic inference for fluke ecology and local transmission foci.

### 4.5. Recommendations for Future Research

To build on these results, future studies should: (1) incorporate molecular diagnostics (PCR/metabarcoding) to enable species-level identification and detect cryptic or zoonotic species; (2) collect standardized management and anthelmintic treatment histories to include as covariates in multivariable models and to assess drug efficacy and resistance risk; (3) link parasitological surveillance to production metrics and clinical assessments to quantify economic impacts of parasitism and inform cost–benefit analyses of control strategies; (4) conduct intermediate host (snail) surveys and environmental monitoring (soil moisture, microclimate) to model trematode transmission risk and guide habitat-targeted interventions; (5) implement longitudinal cohort studies and intervention trials to evaluate seasonally timed control regimens (e.g., strategic deworming prior to monsoon) and their effects on prevalence, intensity and productivity; and (6) establish sentinel monitoring for anthelmintic resistance using fecal egg count reduction tests (FECRT) to guide sustainable drug use policies.

The present study provides a comprehensive epidemiological assessment of gastrointestinal (GI) parasites infecting domestic ruminants in North Bengal, integrating classical prevalence analysis, mixed-effects modeling, and multivariate correspondence approaches [39]. The combination of GLM and MCA yielded a robust framework for identifying host–parasite–environment linkages, highlighting that infection patterns in humid subtropical systems are not stochastic but ecologically structured by host biology, climate, and landscape heterogeneity [61].

### 4.6. Host-Specific Infection Dynamics

The observed variation in infection prevalence across hosts—highest in cattle, followed by sheep and goats—corroborates previous findings that feeding behavior, physiological susceptibility, and immune competence play pivotal roles in parasite transmission. Cattle are predominantly grazers, frequently exposed to fecally contaminated pastures, which explains their stronger association.

In contrast, goats—semi-browsers with a mixed feeding pattern—showed higher representation of Trichuris and *Nematodirus* infections, indicating transmission through drier soil niches and lower exposure to waterborne trematodes. Sheep exhibited a distinctive clustering with Fasciola and *Paramphistomum* spp., two trematode taxa whose transmission depends on Lymnaeid snail populations thriving in waterlogged pasture.

The host-associated infection clusters identified through MCA emphasize niche partitioning of parasites within the domestic ruminant community. Such partitioning likely represents an adaptive strategy that minimizes direct interspecific competition for hosts while maintaining endemic transmission cycles within each species.

## 5. Conclusions

This study provides a comprehensive and statistically assessment of the climatic and host-related determinants shaping gastrointestinal parasite dynamics in domestic ruminants of North Bengal. By integrating GLM, MCA, and heatmap analyses, the research demonstrates that infection patterns are governed by predictable ecological processes rather than random occurrence. Host-specific susceptibility, seasonal climatic variability, and spatial hydro-ecological gradients collectively regulate parasite prevalence, with *Eimeria* spp., *Strongyloides* spp., and *Fasciola* spp. emerging as dominant taxa during humid monsoon and summer periods.

The integration of multivariate and spatial visualization approaches established a quantitative ecological baseline for understanding how biotic and abiotic factors jointly influence parasite transmission. These insights provide critical evidence for developing targeted, host-adapted, and seasonally timed parasite control strategies. Moreover, the findings emphasize the importance of ecosystem-based and One Health–oriented management frameworks to safeguard livestock health, enhance productivity, and strengthen the resilience of agro-pastoral systems under changing climatic conditions.

## Figures and Tables

**Table 1:** Prevalence of gastrointestinal parasites in cattle, goat and sheep across three districts of North Bengal.

Herbivores	Habitat (Different Localities of the North Bengal Districts)	Number of Faecal Samples Collected	Number of Positive	Overall Prevalence (%)
Cattle (N = 518)	Cooch Behar	235	180	76.60
	Alipurduar	214	145	67.76
	Jalpaiguri	69	48	69.57
	Sub total	518	373	72.01
Goat (N = 445)	Cooch Behar	244	162	66.39
	Alipurduar	136	98	72.06
	Jalpaiguri	65	37	56.92
	Sub total	445	297	66.74
Sheep (N = 443)	Cooch Behar	278	194	69.78
	Alipurduar	123	82	66.67
	Jalpaiguri	42	31	73.81
	Sub total	443	307	69.30

**Table 2:** Distribution and prevalence of gastrointestinal parasite infections in domestic herbivores (cattle, goats, and sheep) according to host species, geographic location, season, and parasite taxa in three districts (Cooch Behar, Alipurduar, and Jalpaiguri) of North Bengal, India. Prevalence differences among categories were assessed using the chi-square (χ^2^) test, with corresponding χ^2^ statistics and *p*-values reported.

Characters	Variables	Examined	Positive	Prevalence	χ^2^ Value	*p*-Value
Host Species	Cattle	518	373	72.01	3.14	0.207
	Goat	445	297	66.74		
	Sheep	443	307	69.30		
Area	Cooch Behar	757	536	70.80	1.81	0.40
	Alipurduar	473	325	68.71		
	Jalpaiguri	176	116	65.90		
Season	Summer	536	391	72.95	37.68	0.00
	Monsoon	465	352	75.70		
	Winter	405	234	57.78		
Parasite Species	*Eimeria* spp.	1406	418	29.73	866.74	0.00
	*Fasciola* spp.	1406	212	15.08		
	*Paramphistomum* spp.	1406	178	12.66		
	*Moniezia* spp.	1406	54	3.84		
	*Strongyloides* spp.	1406	253	17.99		
	*Trichostrongylus* spp.	1406	122	8.68		
	*Nematodirus* spp.	1406	76	5.41		
	*Trichuris* spp.	1406	63	4.48		
	*Oxyuris* spp.	1406	42	2.99		

## Data Availability

The datasets used and/or analyzed during the current study are available from the corresponding author on reasonable request.

## Supplementary Materials

The following supporting information can be downloaded at: https://www.mdpi.com/article/10.3390/ani16020338/s1, Supplementary Table S1: General Linear Model results for *Eimeria* spp.; Supplementary Table S2: General Linear Model results for *Fasciola* spp.; Supplementary Table S3: General Linear Model results for *Paramphistomum* spp.; Supplementary Table S4: General Linear Model results for *Moniezia* spp.; Supplementary Table S5: General Linear Model results for *Strongyloides* spp.; Supplementary Table S6: General Linear Model results for *Trichostrongylus* spp.; Supplementary Table S7: General Linear Model results for *Nematodirus* spp.; Supplementary Table S8: General Linear Model results for *Trichuris* spp.; Supplementary Table S9: General Linear Model results for *Oxyuris* spp.; Supplementary Table S10: General Linear Model results for overall gastrointestinal parasite prevalence.

## Abbreviations

The following abbreviations are used in this manuscript:

GI	Gastrointestinal
MCA	Multiple Correspondence Analysis
GLM	Generalised Linear Mixed Model
mL	Milliliter
µL	Microliter
EPG	Eggs Per Gram
OPG	Oocysts Per Gram
Χ^2^	Chi-squared
AIC	Akaike Information Criterion
Dim	Dimension
