# Supplementary material for: Climatic and Host-Related Drivers of Gastrointestinal Parasite Dynamics in Domestic Ruminants of North Bengal, India
Source: Animals (Basel). 2026 Jan 22;16(2):338. doi: 10.3390/ani16020338 (PMC12837427; doi:10.3390/ani16020338)
Supplement: Supplementary file 1 [file animals-16-00338-s001.zip › animals-4005475-supplementary.pdf]

Supplementary Methods: General Linear Model Analysis

To evaluate the influence of host species, season, and geographic area on the prevalence patterns of gastrointestinal parasites, we fitted a series of General Linear Models (GLMs) for each parasite taxon as well as for overall infection status. Presence/absence data for *Eimeria*, *Fasciola*, *Paramphistomum*, *Moniezia*, *Strongyloides*, *Trichostrongylus*, *Nematodirus*, and *Trichuris* spp. were treated as continuous response variables within an ordinary least squares (OLS) framework to ensure comparability across taxa and to accommodate multi-factor interaction structures. For each parasite, the model included Host (cattle, goat, sheep), Season (monsoon, summer, winter), and Area (Alipurduar, Cooch Behar, Jalpaiguri) as fixed effects, together with all two-way and three-way interactions (Host × Season, Host × Area, Season × Area, and Host × Season × Area). Treatment coding was applied for categorical predictors, with cattle, monsoon, and Alipurduar serving as reference levels. Model performance was assessed using R-squared and adjusted R-squared values, and the significance of each factor and interaction term was evaluated using omnibus ANOVA with partial eta squared (η^2p) and omega squared (ω^2) reported as effect size measures. Parameter estimates were presented with standard errors, 95% confidence intervals, and t-tests for inference. All analyses were conducted on the full dataset (n = 1406 observations), and results for each parasite taxon are provided in Supplementary Tables S1–S9.

Supplementary Table S1 – Eimeria spp.

General Linear Model

Model Info

Info	
Estimate	Linear model fit by OLS
Call	Eimeria spp. ~ 1 + Host + Season + Area + Host:Season + Host:Area + Season:Area + Host:Season:Area
R-squared	0.02644
Adj. R-squared	0.00808

Model Results

ANOVA Omnibus tests

	SS	df	F	p	η^2p	ω^2
Model	7.7651	26	1.440	0.071	0.026	0.008
Host	0.3025	2	0.729	0.482	0.001	0.000
Season	0.6468	2	1.560	0.211	0.002	0.001
Area	0.0682	2	0.164	0.848	0.000	0.000
Host ✻ Season	1.4787	4	1.783	0.130	0.005	0.002
Host ✻ Area	0.7597	4	0.916	0.454	0.003	0.000

ANOVA Omnibus tests

	SS	df	F	p	η²p	ω²
Season ✻ Area	1.9264	4	2.322	0.055	0.007	0.004
Host ✻ Season ✻ Area	1.8336	8	1.105	0.357	0.006	0.001
Residuals	285.9646	1379				
Total	293.7297	1405				

Fixed Effects Parameter Estimates

				95% Confidence Interval					
**Names**	**Effect**	**Estimate**	**SE**	**Lower**	**Upper**	**β**	**df**	**t**	**p**
**(Intercept)**	(Intercept)	0.29938	0.0147	0.2705	0.3283	0.0000	1379	20.3353	< .001
**Host1**	Goat - Cattle	0.04299	0.0357	-0.0271	0.1131	0.0940	1379	1.2028	0.229
**Host2**	Sheep - Cattle	0.01676	0.0357	-0.0532	0.0868	0.0367	1379	0.4698	0.639
**Season1**	Summer - Monsoon	0.02825	0.0345	-0.0395	0.0960	0.0618	1379	0.8183	0.413
**Season2**	Winter - Monsoon	-0.03577	0.0373	-0.1090	0.0375	-0.0782	1379	-0.9577	0.338
**Area1**	Cooch Behar - Alipurduar	0.00738	0.0271	-0.0459	0.0606	0.0161	1379	0.2719	0.786
**Area2**	Jalpaiguri - Alipurduar	0.02338	0.0409	-0.0568	0.1036	0.0511	1379	0.5721	0.567
**Host1 ✻ Season1**	Goat - Cattle ✻ Summer - Monsoon	0.01822	0.0838	-0.1461	0.1826	0.0398	1379	0.2174	0.828
**Host2 ✻ Season1**	Sheep - Cattle ✻ Summer - Monsoon	0.05352	0.0828	-0.1088	0.2159	0.1171	1379	0.6468	0.518

**Host1 ✻ Season2**	Goat - Cattle ✻ Winter - Monsoon	0.06122	0.0916	-0.1184	0.2408	0.1339	1379	0.6686	0.504
**Host2 ✻ Season2**	Sheep - Cattle ✻ Winter - Monsoon	-0.12158	0.0901	-0.2984	0.0552	-0.2659	1379	-1.3492	0.177
**Host1 ✻ Area1**	Goat - Cattle ✻ Cooch Behar - Alipurduar	-0.08507	0.0665	-0.2155	0.0453	-0.1861	1379	-1.2798	0.201
**Host2 ✻ Area1**	Sheep - Cattle ✻ Cooch Behar - Alipurduar	-0.10683	0.0648	-0.2340	0.0204	-0.2337	1379	-1.6476	0.100
**Host1 ✻ Area2**	Goat - Cattle ✻ Jalpaiguri - Alipurduar	-0.03691	0.0996	-0.2323	0.1585	-0.0807	1379	-0.3705	0.711
**Host2 ✻ Area2**	Sheep - Cattle ✻ Jalpaiguri - Alipurduar	-0.11658	0.0988	-0.3104	0.0772	-0.2550	1379	-1.1802	0.238
**Season1 ✻ Area1**	Summer - Monsoon ✻ Cooch Behar - Alipurduar	0.15383	0.0643	0.0277	0.2800	0.3364	1379	2.3921	0.017
**Season2 ✻ Area1**	Winter - Monsoon ✻ Cooch Behar - Alipurduar	-0.01178	0.0687	-0.1466	0.1230	-0.0258	1379	-0.1714	0.864
**Season1 ✻ Area2**	Summer - Monsoon ✻ Jalpaigu	0.00975	0.0955	-0.1777	0.1972	0.0213	1379	0.1020	0.919

	ri - Alipurduar								
**Season2 ✻ Area2**	Winter - Monsoon ✻ Jalpaiguri - Alipurduar	-0.02459	0.1039	-0.2285	0.1793	-0.0538	1379	-0.2365	0.813
**Host1 ✻ Season1 ✻ Area1**	Goat - Cattle ✻ Summer - Monsoon ✻ Cooch Behar - Alipurduar	-0.12232	0.1569	-0.4301	0.1855	-0.2675	1379	-0.7795	0.436
**Host2 ✻ Season1 ✻ Area1**	Sheep - Cattle ✻ Summer - Monsoon ✻ Cooch Behar - Alipurduar	0.20501	0.1521	-0.0933	0.5033	0.4484	1379	1.3482	0.178
**Host1 ✻ Season2 ✻ Area1**	Goat - Cattle ✻ Winter - Monsoon ✻ Cooch Behar - Alipurduar	-0.26187	0.1693	-0.5940	0.0703	-0.5727	1379	-1.5465	0.122
**Host2 ✻ Season2 ✻ Area1**	Sheep - Cattle ✻ Winter - Monsoon ✻ Cooch Behar - Alipurduar	-0.00706	0.1643	-0.3294	0.3153	-0.0154	1379	-0.0430	0.966
**Host1 ✻ Season1 ✻ Area2**	Goat - Cattle ✻ Summer - Monsoon ✻	0.00506	0.2334	-0.4528	0.4629	0.0111	1379	0.0217	0.983

	Jalpaiguri - Alipurduar								
**Host2 ✻ Season1 ✻ Area2**	Sheep - Cattle ✻ Summer - Monsoon ✻ Jalpaiguri - Alipurduar	0.41068	0.2276	-0.0359	0.8572	0.8982	1379	1.8042	0.071
**Host1 ✻ Season2 ✻ Area2**	Goat - Cattle ✻ Winter - Monsoon ✻ Jalpaiguri - Alipurduar	-0.05783	0.2553	-0.5587	0.4431	-0.1265	1379	-0.2265	0.821
**Host2 ✻ Season2 ✻ Area2**	Sheep - Cattle ✻ Winter - Monsoon ✻ Jalpaiguri - Alipurduar	0.02698	0.2504	-0.4643	0.5183	0.0590	1379	0.1077	0.914

Supplementary Table S2 – *Fasciola* spp.

General Linear Model

Model Info

Info	
Estimate	Linear model fit by OLS
Call	Fasciola spp. ~ 1 + Host + Season + Area + Host:Season + Host:Area + Season:Area + Host:Season:Area
R-squared	0.0182
Adj. R-squared	-2.75e–4

Model Info

	Info

Model Results

ANOVA Omnibus tests

	SS	df	F	p	η^2p	ω^2
Model	3.283	26	0.985	0.485	0.018	0.000
Host	0.246	2	0.959	0.384	0.001	0.000
Season	0.319	2	1.244	0.289	0.002	0.000
Area	0.159	2	0.620	0.538	0.001	0.000
Host ✻ Season	0.348	4	0.678	0.607	0.002	0.000
Host ✻ Area	1.221	4	2.382	0.050	0.007	0.004
Season ✻ Area	0.774	4	1.510	0.197	0.004	0.001
Host ✻ Season ✻ Area	0.186	8	0.182	0.993	0.001	0.000
Residuals	176.751	1379				
Total	180.034	1405				

Fixed Effects Parameter Estimates

				95% Confidence Interval					
Names	Effect	Estimate	SE	Lower	Upper	β	df	t	p
(Intercept)	(Intercept)	0.15205	0.0116	0.1293	0.1748	0.00000	1379	13.1365	< .001
Host1	Goat - Cattle	-0.00584	0.0281	-0.0610	0.0493	-0.01631	1379	-0.2077	0.835
Host2	Sheep - Cattle	0.03115	0.0281	-0.0239	0.0862	0.08701	1379	1.1103	0.267
Season1	Summer - Monsoon	0.03934	0.0271	-0.0139	0.0926	0.10989	1379	1.4491	0.148

Fixed Effects Parameter Estimates

Names	Effect	Estimate	SE	95% Confidence Interval Lower	95% Confidence Interval Upper	β	df	t	p
Season2	Winter - Monsoon	0.00510	0.0294	-0.0525	0.0627	0.01425	1379	0.1737	0.862
Area1	Cooch Behar - Alipurduar	-0.02212	0.0213	-0.0640	0.0197	-0.06180	1379	-1.0367	0.300
Area2	Jalpaiguri - Alipurduar	-0.00183	0.0321	-0.0649	0.0612	-0.00511	1379	-0.0570	0.955
Host1 ✻ Season1	Goat - Cattle ✻ Summer - Monsoon	-0.01390	0.0659	-0.1431	0.1153	-0.03883	1379	-0.2110	0.833
Host2 ✻ Season1	Sheep - Cattle ✻ Summer - Monsoon	0.03224	0.0651	-0.0954	0.1599	0.09005	1379	0.4955	0.620
Host1 ✻ Season2	Goat - Cattle ✻ Winter - Monsoon	-0.09921	0.0720	-0.2404	0.0420	-0.27716	1379	-1.3782	0.168
Host2 ✻ Season2	Sheep - Cattle ✻ Winter - Monsoon	-0.04273	0.0708	-0.1817	0.0962	-0.11936	1379	-0.6031	0.547
Host1 ✻ Area1	Goat - Cattle ✻ Cooch Behar - Alipurduar	0.08197	0.0523	-0.0205	0.1845	0.22899	1379	1.5685	0.117
Host2 ✻ Area1	Sheep - Cattle ✻ Cooch Behar - Alipurduar	-0.04520	0.0510	-0.1452	0.0548	-0.12626	1379	-0.8866	0.375

Fixed Effects Parameter Estimates

				95% Confidence Interval					
**Names**	**Effect**	**Estimate**	**SE**	**Lower**	**Upper**	**β**	**df**	**t**	**p**
Host1 ✻ Area2	Goat - Cattle ✻ Jalpaiguri - Alipurduar	0.11780	0.0783	-0.0358	0.2714	0.32909	1379	1.5044	0.133
Host2 ✻ Area2	Sheep - Cattle ✻ Jalpaiguri - Alipurduar	0.11327	0.0777	-0.0391	0.2656	0.31644	1379	1.4585	0.145
Season1 ✻ Area1	Summer - Monsoon ✻ Cooch Behar - Alipurduar	0.05306	0.0506	-0.0461	0.1522	0.14822	1379	1.0494	0.294
Season2 ✻ Area1	Winter - Monsoon ✻ Cooch Behar - Alipurduar	0.07717	0.0540	-0.0288	0.1831	0.21558	1379	1.4287	0.153
Season1 ✻ Area2	Summer - Monsoon ✻ Jalpaiguri - Alipurduar	0.13301	0.0751	-0.0143	0.2804	0.37159	1379	1.7708	0.077
Season2 ✻ Area2	Winter - Monsoon ✻ Jalpaiguri - Alipurduar	0.18659	0.0817	0.0263	0.3469	0.52125	1379	2.2833	0.023

Fixed Effects Parameter Estimates

				95% Confidence Interval					
**Names**	**Effect**	**Estimate**	**SE**	**Lower**	**Upper**	**β**	**df**	**t**	**p**
Host1 ✻ Season1 ✻ Area1	Goat - Cattle ✻ Summer - Monsoon ✻ Cooch Behar - Alipurduar	0.07165	0.1234	-0.1703	0.3137	0.20017	1379	0.5808	0.561
Host2 ✻ Season1 ✻ Area1	Sheep - Cattle ✻ Summer - Monsoon ✻ Cooch Behar - Alipurduar	-0.02440	0.1195	-0.2589	0.2101	-0.06817	1379	-0.2041	0.838
Host1 ✻ Season2 ✻ Area1	Goat - Cattle ✻ Winter - Monsoon ✻ Cooch Behar - Alipurduar	0.04902	0.1331	-0.2121	0.3102	0.13693	1379	0.3682	0.713
Host2 ✻ Season2 ✻ Area1	Sheep - Cattle ✻ Winter - Monsoon ✻ Cooch Behar - Alipurduar	-0.01357	0.1292	-0.2670	0.2398	-0.03790	1379	-0.1050	0.916
Host1 ✻ Season1 ✻ Area2	Goat - Cattle ✻ Summer - Monsoon ✻ Jalpaiguri - Alipurduar	0.01271	0.1835	-0.3473	0.3727	0.03549	1379	0.0692	0.945

Fixed Effects Parameter Estimates

				95% Confidence Interval					
Names	Effect	Estimate	SE	Lower	Upper	β	df	t	p
Host2 ✻ Season1 ✻ Area2	Sheep - Cattle ✻ Summer - Monsoon ✻ Jalpaiguri - Alipurduar	-0.06010	0.1790	-0.4112	0.2910	-0.16791	1379	-0.3359	0.737
Host1 ✻ Season2 ✻ Area2	Goat - Cattle ✻ Winter - Monsoon ✻ Jalpaiguri - Alipurduar	-0.10792	0.2007	-0.5017	0.2859	-0.30150	1379	-0.5376	0.591
Host2 ✻ Season2 ✻ Area2	Sheep - Cattle ✻ Winter - Monsoon ✻ Jalpaiguri - Alipurduar	-0.03823	0.1969	-0.4245	0.3480	-0.10679	1379	-0.1941	0.846

Supplementary Table S3 – *Paramphistomum* spp.

General Linear Model

Model Info

Info	
Estimate	Linear model fit by OLS
Call	Paramphistomum spp. ~ 1 + Host + Season + Area + Host:Season + Host:Area + Season:Area + Host:Season:Area

Model Info

Info	
R-squared	0.0324
Adj. R-squared	0.0141

Model Results

ANOVA Omnibus tests

	SS	df	F	p	η^2p	ω^2
Model	5.0313	26	1.774	0.010	0.032	0.014
Host	0.0272	2	0.125	0.883	0.000	0.000
Season	0.2599	2	1.191	0.304	0.002	0.000
Area	0.1104	2	0.506	0.603	0.001	0.000
Host ✻ Season	0.4933	4	1.130	0.340	0.003	0.000
Host ✻ Area	1.9883	4	4.557	0.001	0.013	0.010
Season ✻ Area	0.7514	4	1.722	0.143	0.005	0.002
Host ✻ Season ✻ Area	1.1226	8	1.286	0.246	0.007	0.002
Residuals	150.4338	1379				
Total	155.4651	1405				

Fixed Effects Parameter Estimates

				95% Confidence Interval					
Names	Effect	Estimate	SE	Lower	Upper	β	df	t	p
(Intercept)	(Intercept)	0.12470	0.0107	0.10376	0.14565	0.00000	1379	11.6784	< .001
Host1	Goat - Cattle	0.00467	0.0259	-0.04619	0.05552	0.01403	1379	0.1800	0.857
Host2	Sheep - Cattle	-0.00844	0.0259	-0.05921	0.04233	-0.02537	1379	-0.3260	0.744

Fixed Effects Parameter Estimates

				95% Confidence Interval					
**Names**	**Effect**	**Estimate**	**SE**	**Lower**	**Upper**	**β**	**df**	**t**	**p**
Season1	Summer - Monsoon	0.01059	0.0250	-0.03854	0.05971	0.03183	1379	0.4228	0.673
Season2	Winter - Monsoon	-0.02933	0.0271	-0.08246	0.02381	-0.08817	1379	-1.0828	0.279
Area1	Cooch Behar - Alipurduar	-0.01930	0.0197	-0.05792	0.01932	-0.05803	1379	-0.9806	0.327
Area2	Jalpaiguri - Alipurduar	-0.00590	0.0296	-0.06405	0.05226	-0.01772	1379	-0.1989	0.842
Host1 ✻ Season1	Goat - Cattle ✻ Summer - Monsoon	-0.03597	0.0608	-0.15518	0.08325	-0.10812	1379	-0.5918	0.554
Host2 ✻ Season1	Sheep - Cattle ✻ Summer - Monsoon	-0.11187	0.0600	-0.22961	0.00588	-0.33630	1379	-1.8638	0.063
Host1 ✻ Season2	Goat - Cattle ✻ Winter - Monsoon	0.03582	0.0664	-0.09446	0.16609	0.10767	1379	0.5393	0.590
Host2 ✻ Season2	Sheep - Cattle ✻ Winter - Monsoon	-0.04126	0.0654	-0.16947	0.08695	-0.12404	1379	-0.6313	0.528
Host1 ✻ Area1	Goat - Cattle ✻ Cooch Behar - Alipurduar	-0.09862	0.0482	-0.19319	-0.00404	-0.29646	1379	-2.0454	0.041
Host2 ✻ Area1	Sheep - Cattle ✻ Cooch Behar - Alipurduar	-0.11971	0.0470	-0.21197	-0.02746	-0.35989	1379	-2.5455	0.011

Fixed Effects Parameter Estimates

				95% Confidence Interval					
Names	Effect	Estimate	SE	Lower	Upper	β	df	t	p
Host1 ✻ Area2	Goat - Cattle ✻ Jalpaiguri - Alipurduar	0.13959	0.0722	-0.00213	0.28131	0.41965	1379	1.9323	0.054
Host2 ✻ Area2	Sheep - Cattle ✻ Jalpaiguri - Alipurduar	-0.03885	0.0716	-0.17940	0.10170	-0.11679	1379	-0.5422	0.588
Season1 ✻ Area1	Summer - Monsoon ✻ Cooch Behar - Alipurduar	0.03788	0.0466	-0.05362	0.12938	0.11388	1379	0.8121	0.417
Season2 ✻ Area1	Winter - Monsoon ✻ Cooch Behar - Alipurduar	0.12558	0.0498	0.02782	0.22333	0.37751	1379	2.5201	0.012
Season1 ✻ Area2	Summer - Monsoon ✻ Jalpaiguri - Alipurduar	9.05e-4	0.0693	-0.13504	0.13685	0.00272	1379	0.0131	0.990
Season2 ✻ Area2	Winter - Monsoon ✻ Jalpaiguri - Alipurduar	0.04596	0.0754	-0.10194	0.19385	0.13816	1379	0.6096	0.542

Fixed Effects Parameter Estimates

Names	Effect	Estimate	SE	95% Confidence Interval Lower	95% Confidence Interval Upper	β	df	t	p
Host1 ✻ Season1 ✻ Area1	Goat - Cattle ✻ Summer - Monsoon ✻ Cooch Behar - Alipurduar	-0.16631	0.1138	-0.38957	0.05695	-0.49997	1379	-1.4613	0.144
Host2 ✻ Season1 ✻ Area1	Sheep - Cattle ✻ Summer - Monsoon ✻ Cooch Behar - Alipurduar	-0.08995	0.1103	-0.30630	0.12640	-0.27041	1379	-0.8156	0.415
Host1 ✻ Season2 ✻ Area1	Goat - Cattle ✻ Winter - Monsoon ✻ Cooch Behar - Alipurduar	-0.18694	0.1228	-0.42786	0.05398	-0.56200	1379	-1.5222	0.128
Host2 ✻ Season2 ✻ Area1	Sheep - Cattle ✻ Winter - Monsoon ✻ Cooch Behar - Alipurduar	0.04954	0.1192	-0.18424	0.28331	0.14892	1379	0.4157	0.678
Host1 ✻ Season1 ✻ Area2	Goat - Cattle ✻ Summer - Monsoon ✻ Jalpaiguri - Alipurduar	-0.36397	0.1693	-0.69606	-0.03187	-1.09416	1379	-2.1499	0.032

Fixed Effects Parameter Estimates

Names	Effect	Estimate	SE	95% Confidence Interval Lower	95% Confidence Interval Upper	β	df	t	p
Host2 ✻ Season1 ✻ Area2	Sheep - Cattle ✻ Summer - Monsoon ✻ Jalpaiguri - Alipurduar	-0.24433	0.1651	-0.56821	0.07954	-0.73452	1379	-1.4799	0.139
Host1 ✻ Season2 ✻ Area2	Goat - Cattle ✻ Winter - Monsoon ✻ Jalpaiguri - Alipurduar	-0.07902	0.1852	-0.44230	0.28427	-0.23754	1379	-0.4267	0.670
Host2 ✻ Season2 ✻ Area2	Sheep - Cattle ✻ Winter - Monsoon ✻ Jalpaiguri - Alipurduar	0.04056	0.1816	-0.31578	0.39689	0.12192	1379	0.2233	0.823

Supplementary Table S4 – *Moniezia* spp.

General Linear Model

Model Info

Info	
Estimate	Linear model fit by OLS

Model Info

Info	
Call	Moniezia spp. ~ 1 + Host + Season + Area + Host:Season + Host:Area + Season:Area + Host:Season:Area
R-squared	0.01704
Adj. R-squared	-0.00149

Model Results

ANOVA Omnibus tests

	SS	df	F	p	η²p	ω²
Model	0.88486	26	0.9195	0.581	0.017	0.000
Host	0.00444	2	0.0599	0.942	0.000	0.000
Season	0.01594	2	0.2153	0.806	0.000	0.000
Area	0.14868	2	2.0085	0.135	0.003	0.001
Host ✻ Season	0.02388	4	0.1613	0.958	0.000	0.000
Host ✻ Area	0.17954	4	1.2127	0.304	0.004	0.001
Season ✻ Area	0.05873	4	0.3967	0.811	0.001	0.000
Host ✻ Season ✻ Area	0.39478	8	1.3333	0.222	0.008	0.002
Residuals	51.04117	1379				
Total	51.92603	1405				

Fixed Effects Parameter Estimates

				95% Confidence Interval					
Names	Effect	Estimate	SE	Lower	Upper	β	df	t	p
(Intercept)	(Intercept)	0.03125	0.00622	0.0191	0.04346	0.00000	1379	5.025	< .001
Host1	Goat - Cattle	-0.00223	0.01510	-0.0319	0.02739	-0.01161	1379	-0.148	0.882

Fixed Effects Parameter Estimates

				95% Confidence Interval					
**Names**	**Effect**	**Estimate**	**SE**	**Lower**	**Upper**	**β**	**df**	**t**	**p**
Host2	Sheep - Cattle	-0.00521	0.01508	-0.0348	0.02436	-0.02712	1379	-0.346	0.730
Season1	Summer - Monsoon	0.00897	0.01459	-0.0196	0.03759	0.04667	1379	0.615	0.539
Season2	Winter - Monsoon	0.00165	0.01578	-0.0293	0.03260	0.00858	1379	0.105	0.917
Area1	Cooch Behar - Alipurduar	0.00642	0.01147	-0.0161	0.02891	0.03339	1379	0.560	0.576
Area2	Jalpaiguri - Alipurduar	-0.02631	0.01727	-0.0602	0.00756	-0.13686	1379	-1.524	0.128
Host1 ✻ Season1	Goat - Cattle ✻ Summer - Monsoon	0.00796	0.03540	-0.0615	0.07740	0.04143	1379	0.225	0.822
Host2 ✻ Season1	Sheep - Cattle ✻ Summer - Monsoon	-0.00786	0.03496	-0.0764	0.06072	-0.04090	1379	-0.225	0.822
Host1 ✻ Season2	Goat - Cattle ✻ Winter - Monsoon	0.01098	0.03868	-0.0649	0.08686	0.05709	1379	0.284	0.777
Host2 ✻ Season2	Sheep - Cattle ✻ Winter - Monsoon	-0.01982	0.03807	-0.0945	0.05487	-0.10308	1379	-0.521	0.603
Host1 ✻ Area1	Goat - Cattle ✻ Cooch Behar - Alipurduar	-0.05730	0.02808	-0.1124	-0.00221	-0.29808	1379	-2.040	0.041

Fixed Effects Parameter Estimates

Names	Effect	Estimate	SE	95% Confidence Interval Lower	95% Confidence Interval Upper	β	df	t	p
Host2 ✻ Area1	Sheep - Cattle ✻ Cooch Behar - Alipurduar	-0.01341	0.02739	-0.0671	0.04033	-0.06975	1379	-0.490	0.625
Host1 ✻ Area2	Goat - Cattle ✻ Jalpaiguri - Alipurduar	-0.03572	0.04208	-0.1183	0.04683	-0.18580	1379	-0.849	0.396
Host2 ✻ Area2	Sheep - Cattle ✻ Jalpaiguri - Alipurduar	-0.03014	0.04173	-0.1120	0.05173	-0.15676	1379	-0.722	0.470
Season1 ✻ Area1	Summer - Monsoon ✻ Cooch Behar - Alipurduar	0.02728	0.02717	-0.0260	0.08058	0.14192	1379	1.004	0.315
Season2 ✻ Area1	Winter - Monsoon ✻ Cooch Behar - Alipurduar	0.00966	0.02903	-0.0473	0.06660	0.05024	1379	0.333	0.739
Season1 ✻ Area2	Summer - Monsoon ✻ Jalpaiguri - Alipurduar	0.02102	0.04037	-0.0582	0.10020	0.10932	1379	0.521	0.603
Season2 ✻ Area2	Winter - Monsoon ✻ Jalpaiguri - Alipurduar	0.03361	0.04391	-0.0525	0.11975	0.17481	1379	0.765	0.444

Fixed Effects Parameter Estimates

				95% Confidence Interval					
Names	Effect	Estimate	SE	Lower	Upper	β	df	t	p
Host1 ✻ Season1 ✻ Area1	Goat - Cattle ✻ Summer - Monsoon ✻ Cooch Behar - Alipurduar	-0.03788	0.06629	-0.1679	0.09216	-0.19705	1379	-0.571	0.568
Host2 ✻ Season1 ✻ Area1	Sheep - Cattle ✻ Summer - Monsoon ✻ Cooch Behar - Alipurduar	0.07896	0.06424	-0.0471	0.20498	0.41073	1379	1.229	0.219
Host1 ✻ Season2 ✻ Area1	Goat - Cattle ✻ Winter - Monsoon ✻ Cooch Behar - Alipurduar	-0.11401	0.07154	-0.2543	0.02632	-0.59307	1379	-1.594	0.111
Host2 ✻ Season2 ✻ Area1	Sheep - Cattle ✻ Winter - Monsoon ✻ Cooch Behar - Alipurduar	0.08223	0.06942	-0.0539	0.21841	0.42776	1379	1.185	0.236
Host1 ✻ Season1 ✻ Area2	Goat - Cattle ✻ Summer - Monsoon ✻ Jalpaiguri - Alipurduar	0.03161	0.09861	-0.1618	0.22506	0.16444	1379	0.321	0.749

Fixed Effects Parameter Estimates

Names	Effect	Estimate	SE	95% Confidence Interval Lower	95% Confidence Interval Upper	β	df	t	p
Host2 ✻ Season1 ✻ Area2	Sheep - Cattle ✻ Summer - Monsoon ✻ Jalpaiguri - Alipurduar	0.05128	0.09617	-0.1374	0.23993	0.26672	1379	0.533	0.594
Host1 ✻ Season2 ✻ Area2	Goat - Cattle ✻ Winter - Monsoon ✻ Jalpaiguri - Alipurduar	-0.16722	0.10787	-0.3788	0.04439	-0.86983	1379	-1.550	0.121
Host2 ✻ Season2 ✻ Area2	Sheep - Cattle ✻ Winter - Monsoon ✻ Jalpaiguri - Alipurduar	-0.02291	0.10581	-0.2305	0.18465	-0.11916	1379	-0.217	0.829

Supplementary Table S5 – *Strongyloides* spp.

General Linear Model

Model Info

Info	
Estimate	Linear model fit by OLS
Call	Strongyloides spp. ~ 1 + Host + Season + Area + Host:Season + Host:Area + Season:Area + Host:Season:Area

Model Info

Info	
R-squared	0.01955
Adj. R-squared	0.00106

Model Results

ANOVA Omnibus tests

	SS	df	F	p	η^2p	ω^2
Model	4.056	26	1.057	0.386	0.020	0.001
Host	0.601	2	2.037	0.131	0.003	0.001
Season	0.727	2	2.464	0.086	0.004	0.002
Area	0.168	2	0.570	0.565	0.001	0.000
Host ✻ Season	0.694	4	1.176	0.319	0.003	0.001
Host ✻ Area	0.225	4	0.381	0.822	0.001	0.000
Season ✻ Area	0.137	4	0.233	0.920	0.001	0.000
Host ✻ Season ✻ Area	1.959	8	1.660	0.104	0.010	0.004
Residuals	203.419	1379				
Total	207.474	1405				

Fixed Effects Parameter Estimates

				95% Confidence Interval					
Names	Effect	Estimate	SE	Lower	Upper	β	df	t	p
(Intercept)	(Intercept)	0.18327	0.0124	0.15892	0.20763	0.0000	1379	14.760	< .001
Host1	Goat - Cattle	-0.05588	0.0301	-0.11502	0.00326	-0.1454	1379	-1.854	0.064
Host2	Sheep - Cattle	-0.00504	0.0301	-0.06407	0.05400	-0.0131	1379	-0.167	0.867

Fixed Effects Parameter Estimates

Names	Effect	Estimate	SE	95% Confidence Interval Lower	95% Confidence Interval Upper	β	df	t	p
Season1	Summer - Monsoon	0.05371	0.0291	-0.00341	0.11084	0.1398	1379	1.844	0.065
Season2	Winter - Monsoon	-0.00517	0.0315	-0.06696	0.05661	-0.0135	1379	-0.164	0.870
Area1	Cooch Behar - Alipurduar	0.00828	0.0229	-0.03663	0.05319	0.0215	1379	0.362	0.718
Area2	Jalpaiguri - Alipurduar	0.03670	0.0345	-0.03092	0.10432	0.0955	1379	1.065	0.287
Host1 ✻ Season1	Goat - Cattle ✻ Summer - Monsoon	0.15020	0.0707	0.01158	0.28883	0.3909	1379	2.126	0.034
Host2 ✻ Season1	Sheep - Cattle ✻ Summer - Monsoon	0.08889	0.0698	-0.04803	0.22580	0.2313	1379	1.274	0.203
Host1 ✻ Season2	Goat - Cattle ✻ Winter - Monsoon	0.06766	0.0772	-0.08383	0.21915	0.1761	1379	0.876	0.381
Host2 ✻ Season2	Sheep - Cattle ✻ Winter - Monsoon	0.04519	0.0760	-0.10390	0.19429	0.1176	1379	0.595	0.552
Host1 ✻ Area1	Goat - Cattle ✻ Cooch Behar - Alipurduar	-0.02459	0.0561	-0.13457	0.08539	-0.0640	1379	-0.439	0.661
Host2 ✻ Area1	Sheep - Cattle ✻ Cooch Behar - Alipurduar	-0.04145	0.0547	-0.14873	0.06583	-0.1079	1379	-0.758	0.449

Fixed Effects Parameter Estimates

Names	Effect	Estimate	SE	95% Confidence Interval Lower	95% Confidence Interval Upper	β	df	t	p
Host1 ✻ Area2	Goat - Cattle ✻ Jalpaiguri - Alipurduar	-0.08808	0.0840	-0.25288	0.07672	-0.2292	1379	-1.048	0.295
Host2 ✻ Area2	Sheep - Cattle ✻ Jalpaiguri - Alipurduar	-0.04967	0.0833	-0.21311	0.11377	-0.1293	1379	-0.596	0.551
Season1 ✻ Area1	Summer - Monsoon ✻ Cooch Behar - Alipurduar	-0.04114	0.0542	-0.14754	0.06526	-0.1071	1379	-0.758	0.448
Season2 ✻ Area1	Winter - Monsoon ✻ Cooch Behar - Alipurduar	-0.02363	0.0579	-0.13730	0.09004	-0.0615	1379	-0.408	0.683
Season1 ✻ Area2	Summer - Monsoon ✻ Jalpaiguri - Alipurduar	0.01551	0.0806	-0.14257	0.17359	0.0404	1379	0.192	0.847
Season2 ✻ Area2	Winter - Monsoon ✻ Jalpaiguri - Alipurduar	0.02192	0.0877	-0.15005	0.19390	0.0571	1379	0.250	0.803

Fixed Effects Parameter Estimates

				95% Confidence Interval					
Names	Effect	Estimate	SE	Lower	Upper	β	df	t	p
Host1 ✻ Season1 ✻ Area1	Goat - Cattle ✻ Summer - Monsoon ✻ Cooch Behar - Alipurduar	-0.13845	0.1323	-0.39807	0.12117	-0.3603	1379	-1.046	0.296
Host2 ✻ Season1 ✻ Area1	Sheep - Cattle ✻ Summer - Monsoon ✻ Cooch Behar - Alipurduar	0.25524	0.1283	0.00365	0.50683	0.6642	1379	1.990	0.047
Host1 ✻ Season2 ✻ Area1	Goat - Cattle ✻ Winter - Monsoon ✻ Cooch Behar - Alipurduar	-0.08446	0.1428	-0.36461	0.19569	-0.2198	1379	-0.591	0.554
Host2 ✻ Season2 ✻ Area1	Sheep - Cattle ✻ Winter - Monsoon ✻ Cooch Behar - Alipurduar	0.26999	0.1386	-0.00185	0.54184	0.7026	1379	1.948	0.052
Host1 ✻ Season1 ✻ Area2	Goat - Cattle ✻ Summer - Monsoon ✻ Jalpaiguri - Alipurduar	0.10951	0.1969	-0.27667	0.49569	0.2850	1379	0.556	0.578

Fixed Effects Parameter Estimates

Names	Effect	Estimate	SE	95% Confidence Interval Lower	95% Confidence Interval Upper	β	df	t	p
Host2 ✻ Season1 ✻ Area2	Sheep - Cattle ✻ Summer - Monsoon ✻ Jalpaiguri - Alipurduar	0.29201	0.1920	-0.08461	0.66863	0.7599	1379	1.521	0.128
Host1 ✻ Season2 ✻ Area2	Goat - Cattle ✻ Winter - Monsoon ✻ Jalpaiguri - Alipurduar	0.14054	0.2153	-0.28191	0.56298	0.3657	1379	0.653	0.514
Host2 ✻ Season2 ✻ Area2	Sheep - Cattle ✻ Winter - Monsoon ✻ Jalpaiguri - Alipurduar	0.44894	0.2112	0.03458	0.86331	1.1683	1379	2.125	0.034

Supplementary Table S6 – *Trichostrongylus* spp.

General Linear Model

Model Info

Info	
Estimate	Linear model fit by OLS

Model Info

Info	
Call	Trichostrongylus spp. ~ 1 + Host + Season + Area + Host:Season + Host:Area + Season:Area + Host:Season:Area
R-squared	0.0187
Adj. R-squared	2.48e-4

Model Results

ANOVA Omnibus tests

	SS	df	F	p	η^2_p	ω^2
Model	2.07336	26	1.0134	0.445	0.019	0.000
Host	0.13564	2	0.8619	0.423	0.001	0.000
Season	0.00683	2	0.0434	0.958	0.000	0.000
Area	0.00917	2	0.0583	0.943	0.000	0.000
Host ✻ Season	0.17227	4	0.5473	0.701	0.002	0.000
Host ✻ Area	0.54231	4	1.7229	0.142	0.005	0.002
Season ✻ Area	0.54658	4	1.7365	0.139	0.005	0.002
Host ✻ Season ✻ Area	0.43128	8	0.6851	0.705	0.004	0.000
Residuals	108.51341	1379				
Total	110.58677	1405				

Fixed Effects Parameter Estimates

				95% Confidence Interval					
Names	Effect	Estimate	SE	Lower	Upper	β	df	t	p
(Intercept)	(Intercept)	0.08515	0.00907	0.0674	0.10294	0.00000	1379	9.3894	< .001
Host1	Goat - Cattle	-0.02866	0.02202	-0.0719	0.01454	-0.10214	1379	-1.3015	0.193

Fixed Effects Parameter Estimates

Names	Effect	Estimate	SE	95% Confidence Interval		β	df	t	p
				Lower	Upper				
Host2	Sheep - Cattle	-0.01011	0.02198	-0.0532	0.03301	-0.03603	1379	-0.4599	0.646
Season1	Summer - Monsoon	0.00615	0.02127	-0.0356	0.04787	0.02191	1379	0.2890	0.773
Season2	Winter - Monsoon	0.00212	0.02300	-0.0430	0.04725	0.00756	1379	0.0922	0.927
Area1	Cooch Behar - Alipurduar	-0.00249	0.01672	-0.0353	0.03031	-0.00889	1379	-0.1492	0.881
Area2	Jalpaiguri - Alipurduar	0.00550	0.02518	-0.0439	0.05489	0.01960	1379	0.2184	0.827
Host1 ✻ Season1	Goat - Cattle ✻ Summer - Monsoon	0.05469	0.05161	-0.0466	0.15594	0.19492	1379	1.0595	0.290
Host2 ✻ Season1	Sheep - Cattle ✻ Summer - Monsoon	0.01904	0.05098	-0.0810	0.11904	0.06788	1379	0.3736	0.709
Host1 ✻ Season2	Goat - Cattle ✻ Winter - Monsoon	0.07519	0.05640	-0.0355	0.18584	0.26802	1379	1.3331	0.183
Host2 ✻ Season2	Sheep - Cattle ✻ Winter - Monsoon	0.01298	0.05551	-0.0959	0.12187	0.04626	1379	0.2338	0.815
Host1 ✻ Area1	Goat - Cattle ✻ Cooch Behar - Alipurduar	0.03182	0.04095	-0.0485	0.11215	0.11343	1379	0.7772	0.437

Fixed Effects Parameter Estimates

				95% Confidence Interval					
Names	Effect	Estimate	SE	Lower	Upper	β	df	t	p
Host2 ✻ Area1	Sheep - Cattle ✻ Cooch Behar - Alipurduar	-0.03618	0.03994	-0.1145	0.04218	-0.12895	1379	-0.9058	0.365
Host1 ✻ Area2	Goat - Cattle ✻ Jalpaiguri - Alipurduar	-0.04544	0.06136	-0.1658	0.07492	-0.16198	1379	-0.7406	0.459
Host2 ✻ Area2	Sheep - Cattle ✻ Jalpaiguri - Alipurduar	-0.13538	0.06085	-0.2548	-0.01601	-0.48255	1379	-2.2248	0.026
Season1 ✻ Area1	Summer - Monsoon ✻ Cooch Behar - Alipurduar	-0.06962	0.03961	-0.1473	0.00809	-0.24815	1379	-1.7574	0.079
Season2 ✻ Area1	Winter - Monsoon ✻ Cooch Behar - Alipurduar	-0.01375	0.04232	-0.0968	0.06927	-0.04901	1379	-0.3249	0.745
Season1 ✻ Area2	Summer - Monsoon ✻ Jalpaiguri - Alipurduar	-0.10172	0.05886	-0.2172	0.01373	-0.36258	1379	-1.7283	0.084
Season2 ✻ Area2	Winter - Monsoon ✻ Jalpaiguri - Alipurduar	0.03548	0.06403	-0.0901	0.16109	0.12648	1379	0.5542	0.580

Fixed Effects Parameter Estimates

Names	Effect	Estimate	SE	95% Confidence Interval Lower	95% Confidence Interval Upper	β	df	t	p
Host1 ✻ Season1 ✻ Area1	Goat - Cattle ✻ Summer - Monsoon ✻ Cooch Behar - Alipurduar	0.10964	0.09666	-0.0800	0.29926	0.39079	1379	1.1343	0.257
Host2 ✻ Season1 ✻ Area1	Sheep - Cattle ✻ Summer - Monsoon ✻ Cooch Behar - Alipurduar	0.00911	0.09367	-0.1746	0.19286	0.03247	1379	0.0973	0.923
Host1 ✻ Season2 ✻ Area1	Goat - Cattle ✻ Winter - Monsoon ✻ Cooch Behar - Alipurduar	0.07759	0.10431	-0.1270	0.28221	0.27656	1379	0.7439	0.457
Host2 ✻ Season2 ✻ Area1	Sheep - Cattle ✻ Winter - Monsoon ✻ Cooch Behar - Alipurduar	0.05488	0.10121	-0.1437	0.25343	0.19561	1379	0.5422	0.588
Host1 ✻ Season1 ✻ Area2	Goat - Cattle ✻ Summer - Monsoon ✻ Jalpaiguri - Alipurduar	0.12802	0.14378	-0.1540	0.41007	0.45630	1379	0.8904	0.373

Fixed Effects Parameter Estimates

Names	Effect	Estimate	SE	95% Confidence Interval Lower	95% Confidence Interval Upper	β	df	t	p
Host2 ✻ Season1 ✻ Area2	Sheep - Cattle ✻ Summer - Monsoon ✻ Jalpaiguri - Alipurduar	-0.07414	0.14022	-0.3492	0.20093	-0.26426	1379	-0.5287	0.597
Host1 ✻ Season2 ✻ Area2	Goat - Cattle ✻ Winter - Monsoon ✻ Jalpaiguri - Alipurduar	0.27594	0.15728	-0.0326	0.58448	0.98355	1379	1.7544	0.080
Host2 ✻ Season2 ✻ Area2	Sheep - Cattle ✻ Winter - Monsoon ✻ Jalpaiguri - Alipurduar	0.05034	0.15428	-0.2523	0.35298	0.17943	1379	0.3263	0.744

Supplementary Table S7 – *Nematodirus* spp.

General Linear Model

Model Info

Info	
Estimate	Linear model fit by OLS
Call	Nematodirus spp. ~ 1 + Host + Season + Area + Host:Season + Host:Area + Season:Area + Host:Season:Area

Model Info

Info	
R-squared	0.02088
Adj. R-squared	0.00242

Model Results

ANOVA Omnibus tests

	SS	df	F	p	η^2p	ω^2
Model	1.5009	26	1.131	0.296	0.021	0.002
Host	0.0835	2	0.818	0.442	0.001	0.000
Season	0.0193	2	0.189	0.828	0.000	0.000
Area	0.0842	2	0.825	0.439	0.001	0.000
Host ✻ Season	0.1104	4	0.541	0.706	0.002	0.000
Host ✻ Area	0.1584	4	0.776	0.541	0.002	0.000
Season ✻ Area	0.0516	4	0.253	0.908	0.001	0.000
Host ✻ Season ✻ Area	0.8613	8	2.109	0.032	0.012	0.006
Residuals	70.3910	1379				
Total	71.8919	1405				

Fixed Effects Parameter Estimates

				95% Confidence Interval					
Names	Effect	Estimate	SE	Lower	Upper	β	df	t	p
(Intercept)	(Intercept)	0.04956	0.00730	0.0352	0.0639	0.00000	1379	6.7854	< .001
Host1	Goat - Cattle	0.02146	0.01773	-0.0133	0.0562	0.09486	1379	1.2100	0.226
Host2	Sheep - Cattle	0.00361	0.01770	-0.0311	0.0383	0.01597	1379	0.2041	0.838

Fixed Effects Parameter Estimates

Names	Effect	Estimate	SE	95% Confidence Interval		β	df	t	p
				Lower	Upper				
Season1	Summer - Monsoon	-0.00212	0.01713	-0.0357	0.0315	-0.00935	1379	-0.1235	0.902
Season2	Winter - Monsoon	-0.01084	0.01853	-0.0472	0.0255	-0.04791	1379	-0.5849	0.559
Area1	Cooch Behar - Alipurduar	-0.00630	0.01347	-0.0327	0.0201	-0.02787	1379	-0.4682	0.640
Area2	Jalpaiguri - Alipurduar	-0.02601	0.02028	-0.0658	0.0138	-0.11497	1379	-1.2826	0.200
Host1 ✻ Season1	Goat - Cattle ✻ Summer - Monsoon	-0.03603	0.04157	-0.1176	0.0455	-0.15928	1379	-0.8667	0.386
Host2 ✻ Season1	Sheep - Cattle ✻ Summer - Monsoon	-0.00280	0.04106	-0.0833	0.0777	-0.01236	1379	-0.0681	0.946
Host1 ✻ Season2	Goat - Cattle ✻ Winter - Monsoon	-0.03935	0.04543	-0.1285	0.0498	-0.17394	1379	-0.8661	0.387
Host2 ✻ Season2	Sheep - Cattle ✻ Winter - Monsoon	0.02383	0.04471	-0.0639	0.1115	0.10533	1379	0.5329	0.594
Host1 ✻ Area1	Goat - Cattle ✻ Cooch Behar - Alipurduar	0.00201	0.03298	-0.0627	0.0667	0.00890	1379	0.0611	0.951
Host2 ✻ Area1	Sheep - Cattle ✻ Cooch Behar - Alipurduar	0.01140	0.03217	-0.0517	0.0745	0.05041	1379	0.3545	0.723

Fixed Effects Parameter Estimates

				95% Confidence Interval					
Names	Effect	Estimate	SE	Lower	Upper	β	df	t	p
Host1 ✻ Area2	Goat - Cattle ✻ Jalpaiguri - Alipurduar	0.01533	0.04942	-0.0816	0.1123	0.06778	1379	0.3103	0.756
Host2 ✻ Area2	Sheep - Cattle ✻ Jalpaiguri - Alipurduar	-0.05369	0.04901	-0.1498	0.0424	-0.23737	1379	-1.0956	0.273
Season1 ✻ Area1	Summer - Monsoon ✻ Cooch Behar - Alipurduar	0.02063	0.03191	-0.0420	0.0832	0.09120	1379	0.6466	0.518
Season2 ✻ Area1	Winter - Monsoon ✻ Cooch Behar - Alipurduar	0.02030	0.03409	-0.0466	0.0872	0.08973	1379	0.5955	0.552
Season1 ✻ Area2	Summer - Monsoon ✻ Jalpaiguri - Alipurduar	0.00301	0.04740	-0.0900	0.0960	0.01333	1379	0.0636	0.949
Season2 ✻ Area2	Winter - Monsoon ✻ Jalpaiguri - Alipurduar	0.03493	0.05157	-0.0662	0.1361	0.15441	1379	0.6773	0.498

Fixed Effects Parameter Estimates

Names	Effect	Estimate	SE	95% Confidence Interval Lower	95% Confidence Interval Upper	β	df	t	p
Host1 ✻ Season1 ✻ Area1	Goat - Cattle ✻ Summer - Monsoon ✻ Cooch Behar - Alipurduar	0.16861	0.07785	0.0159	0.3213	0.74540	1379	2.1658	0.030
Host2 ✻ Season1 ✻ Area1	Sheep - Cattle ✻ Summer - Monsoon ✻ Cooch Behar - Alipurduar	-0.07553	0.07544	-0.2235	0.0725	-0.33392	1379	-1.0012	0.317
Host1 ✻ Season2 ✻ Area1	Goat - Cattle ✻ Winter - Monsoon ✻ Cooch Behar - Alipurduar	0.04794	0.08401	-0.1169	0.2127	0.21193	1379	0.5706	0.568
Host2 ✻ Season2 ✻ Area1	Sheep - Cattle ✻ Winter - Monsoon ✻ Cooch Behar - Alipurduar	-0.19528	0.08152	-0.3552	-0.0354	-0.86327	1379	-2.3955	0.017
Host1 ✻ Season1 ✻ Area2	Goat - Cattle ✻ Summer - Monsoon ✻ Jalpaiguri - Alipurduar	0.24330	0.11580	0.0161	0.4705	1.07556	1379	2.1010	0.036

Fixed Effects Parameter Estimates

				95% Confidence Interval					
Names	Effect	Estimate	SE	Lower	Upper	β	df	t	p
Host2 ✻ Season1 ✻ Area2	Sheep - Cattle ✻ Summer - Monsoon ✻ Jalpaiguri - Alipurduar	0.03461	0.11294	-0.1869	0.2562	0.15300	1379	0.3065	0.759
Host1 ✻ Season2 ✻ Area2	Goat - Cattle ✻ Winter - Monsoon ✻ Jalpaiguri - Alipurduar	0.05742	0.12668	-0.1911	0.3059	0.25384	1379	0.4533	0.650
Host2 ✻ Season2 ✻ Area2	Sheep - Cattle ✻ Winter - Monsoon ✻ Jalpaiguri - Alipurduar	-0.15895	0.12426	-0.4027	0.0848	-0.70267	1379	-1.2792	0.201

Supplementary Table S8 – *Trichuris* spp.

Model Info

Info	
Estimate	Linear model fit by OLS
Call	Trichuris spp. ~ 1 + Host + Season + Area + Host:Season + Host:Area + Season:Area + Host:Season:Area
R-squared	0.02392

Model Info

Info	
Adj. R-squared	0.00551

Model Results

ANOVA Omnibus tests

	SS	df	F	p	η^2p	ω^2
Model	1.4393	26	1.300	0.143	0.024	0.006
Host	0.0782	2	0.918	0.400	0.001	0.000
Season	0.0811	2	0.951	0.386	0.001	0.000
Area	0.0785	2	0.921	0.398	0.001	0.000
Host ✻ Season	0.2921	4	1.714	0.144	0.005	0.002
Host ✻ Area	0.1850	4	1.086	0.362	0.003	0.000
Season ✻ Area	0.5105	4	2.996	0.018	0.009	0.006
Host ✻ Season ✻ Area	0.5127	8	1.505	0.151	0.009	0.003
Residuals	58.7378	1379				
Total	60.1771	1405				

Fixed Effects Parameter Estimates

				95% Confidence Interval					
Names	Effect	Estimate	SE	Lower	Upper	β	df	t	p
(Intercept)	(Intercept)	0.04441	0.00667	0.03132	0.0575	0.00000	1379	6.6563	< .001
Host1	Goat - Cattle	0.02092	0.01620	-0.01085	0.0527	0.10110	1379	1.2916	0.197
Host2	Sheep - Cattle	0.00400	0.01617	-0.02773	0.0357	0.01931	1379	0.2471	0.805
Season1	Summer - Monsoon	-0.01871	0.01565	-0.04941	0.0120	-0.09040	1379	-1.1956	0.232

Fixed Effects Parameter Estimates

Names	Effect	Estimate	SE	95% Confidence Interval Lower	95% Confidence Interval Upper	β	df	t	p
Season2	Winter - Monsoon	-0.02011	0.01693	-0.05332	0.0131	-0.09719	1379	-1.1884	0.235
Area1	Cooch Behar - Alipurduar	0.01551	0.01230	-0.00862	0.0396	0.07495	1379	1.2610	0.208
Area2	Jalpaiguri - Alipurduar	0.01809	0.01852	-0.01825	0.0544	0.08739	1379	0.9764	0.329
Host1 ✻ Season1	Goat - Cattle ✻ Summer - Monsoon	-0.03925	0.03797	-0.11374	0.0352	-0.18965	1379	-1.0336	0.301
Host2 ✻ Season1	Sheep - Cattle ✻ Summer - Monsoon	-6.85e−4	0.03750	-0.07426	0.0729	-0.00331	1379	-0.0183	0.985
Host1 ✻ Season2	Goat - Cattle ✻ Winter - Monsoon	-0.04292	0.04150	-0.12433	0.0385	-0.20740	1379	-1.0343	0.301
Host2 ✻ Season2	Sheep - Cattle ✻ Winter - Monsoon	0.06063	0.04084	-0.01949	0.1407	0.29294	1379	1.4845	0.138
Host1 ✻ Area1	Goat - Cattle ✻ Cooch Behar - Alipurduar	0.03725	0.03013	-0.02185	0.0963	0.17999	1379	1.2364	0.217
Host2 ✻ Area1	Sheep - Cattle ✻ Cooch Behar - Alipurduar	0.01567	0.02939	-0.04198	0.0733	0.07572	1379	0.5333	0.594

Fixed Effects Parameter Estimates

				95% Confidence Interval					
**Names**	**Effect**	**Estimate**	**SE**	**Lower**	**Upper**	**β**	**df**	**t**	**p**
Host1 ✻ Area2	Goat - Cattle ✻ Jalpaiguri - Alipurduar	0.08216	0.04514	-0.00640	0.1707	0.39698	1379	1.8200	0.069
Host2 ✻ Area2	Sheep - Cattle ✻ Jalpaiguri - Alipurduar	0.06959	0.04477	-0.01823	0.1574	0.33626	1379	1.5544	0.120
Season1 ✻ Area1	Summer - Monsoon ✻ Cooch Behar - Alipurduar	-0.00678	0.02915	-0.06395	0.0504	-0.03276	1379	-0.2326	0.816
Season2 ✻ Area1	Winter - Monsoon ✻ Cooch Behar - Alipurduar	0.00598	0.03114	-0.05510	0.0671	0.02890	1379	0.1921	0.848
Season1 ✻ Area2	Summer - Monsoon ✻ Jalpaiguri - Alipurduar	-0.13951	0.04330	-0.22446	-0.0546	-0.67411	1379	-3.2218	0.001
Season2 ✻ Area2	Winter - Monsoon ✻ Jalpaiguri - Alipurduar	-0.06436	0.04711	-0.15677	0.0281	-0.31096	1379	-1.3661	0.172

Fixed Effects Parameter Estimates

				95% Confidence Interval					
**Names**	**Effect**	**Estimate**	**SE**	**Lower**	**Upper**	**β**	**df**	**t**	**p**
Host1 ✻ Season1 ✻ Area1	Goat - Cattle ✻ Summer - Monsoon ✻ Cooch Behar - Alipurduar	-0.01937	0.07112	-0.15888	0.1201	-0.09360	1379	-0.2724	0.785
Host2 ✻ Season1 ✻ Area1	Sheep - Cattle ✻ Summer - Monsoon ✻ Cooch Behar - Alipurduar	0.01186	0.06892	-0.12333	0.1471	0.05731	1379	0.1721	0.863
Host1 ✻ Season2 ✻ Area1	Goat - Cattle ✻ Winter - Monsoon ✻ Cooch Behar - Alipurduar	-0.04454	0.07674	-0.19509	0.1060	-0.21523	1379	-0.5804	0.562
Host2 ✻ Season2 ✻ Area1	Sheep - Cattle ✻ Winter - Monsoon ✻ Cooch Behar - Alipurduar	-0.01373	0.07447	-0.15980	0.1324	-0.06632	1379	-0.1843	0.854
Host1 ✻ Season1 ✻ Area2	Goat - Cattle ✻ Summer - Monsoon ✻ Jalpaiguri - Alipurduar	-0.23557	0.10578	-0.44309	-0.0281	-1.13827	1379	-2.2269	0.026

Fixed Effects Parameter Estimates

				95% Confidence Interval					
**Names**	**Effect**	**Estimate**	**SE**	**Lower**	**Upper**	**β**	**df**	**t**	**p**
Host2 ✻ Season1 ✻ Area2	Sheep - Cattle ✻ Summer - Monsoon ✻ Jalpaiguri - Alipurduar	0.00371	0.10317	-0.19867	0.2061	0.01793	1379	0.0360	0.971
Host1 ✻ Season2 ✻ Area2	Goat - Cattle ✻ Winter - Monsoon ✻ Jalpaiguri - Alipurduar	-0.24265	0.11572	-0.46965	-0.0156	-1.17245	1379	-2.0969	0.036
Host2 ✻ Season2 ✻ Area2	Sheep - Cattle ✻ Winter - Monsoon ✻ Jalpaiguri - Alipurduar	0.09895	0.11351	-0.12371	0.3216	0.47813	1379	0.8718	0.383

Supplementary Table S9– *Oxyuris* spp.

General Linear Model

Model Info

**Info**	
Estimate	Linear model fit by OLS
Call	Oxyuris spp. ~ 1 + Host + Season + Area + Host:Season + Host:Area + Season:Area + Host:Season:Area
R-squared	0.0183

Model Info

Info	
Adj. R-squared	-2.01e−4

Model Results

ANOVA Omnibus tests

	SS	df	F	p	η²p	ω²
Model	0.74599	26	0.9892	0.480	0.018	0.000
Host	7.23e-4	2	0.0125	0.988	0.000	0.000
Season	0.05655	2	0.9747	0.378	0.001	0.000
Area	0.00222	2	0.0383	0.962	0.000	0.000
Host ✻ Season	0.06480	4	0.5585	0.693	0.002	0.000
Host ✻ Area	0.16179	4	1.3944	0.234	0.004	0.001
Season ✻ Area	0.14350	4	1.2368	0.293	0.004	0.001
Host ✻ Season ✻ Area	0.30217	8	1.3022	0.238	0.007	0.002
Residuals	39.99939	1379				
Total	40.74538	1405				

Fixed Effects Parameter Estimates

				95% Confidence Interval					
Names	Effect	Estimate	SE	Lower	Upper	β	df	t	p
(Intercept)	(Intercept)	0.02984	0.00551	0.01903	0.04064	0.00000	1379	5.4187	< .001
Host1	Goat - Cattle	-8.62e−4	0.01337	-0.02709	0.02536	-0.00506	1379	-0.0645	0.949
Host2	Sheep - Cattle	0.00129	0.01335	-0.02489	0.02747	0.00759	1379	0.0968	0.923
Season1	Summer - Monsoon	0.01798	0.01291	-0.00735	0.04331	0.10558	1379	1.3923	0.164

Fixed Effects Parameter Estimates

				95% Confidence Interval					
**Names**	**Effect**	**Estimate**	**SE**	**Lower**	**Upper**	**β**	**df**	**t**	**p**
Season2	Winter - Monsoon	0.00832	0.01397	-0.01908	0.03571	0.04883	1379	0.5954	0.552
Area1	Cooch Behar - Alipurduar	-0.00281	0.01015	-0.02272	0.01711	-0.01648	1379	-0.2764	0.782
Area2	Jalpaiguri - Alipurduar	-0.00197	0.01529	-0.03195	0.02802	-0.01156	1379	-0.1288	0.898
Host1 ✻ Season1	Goat - Cattle ✻ Summer - Monsoon	0.00203	0.03134	-0.05944	0.06350	0.01194	1379	0.0649	0.948
Host2 ✻ Season1	Sheep - Cattle ✻ Summer - Monsoon	0.01054	0.03095	-0.05017	0.07126	0.06192	1379	0.3407	0.733
Host1 ✻ Season2	Goat - Cattle ✻ Winter - Monsoon	-0.02814	0.03424	-0.09531	0.03904	-0.16522	1379	-0.8216	0.411
Host2 ✻ Season2	Sheep - Cattle ✻ Winter - Monsoon	0.01870	0.03370	-0.04741	0.08481	0.10981	1379	0.5549	0.579
Host1 ✻ Area1	Goat - Cattle ✻ Cooch Behar - Alipurduar	-0.04237	0.02486	-0.09114	0.00640	-0.24880	1379	-1.7042	0.089
Host2 ✻ Area1	Sheep - Cattle ✻ Cooch Behar - Alipurduar	0.01166	0.02425	-0.03592	0.05923	0.06845	1379	0.4807	0.631

Fixed Effects Parameter Estimates

				95% Confidence Interval					
Names	Effect	Estimate	SE	Lower	Upper	β	df	t	p
Host1 ✻ Area2	Goat - Cattle ✻ Jalpaiguri - Alipurduar	-0.00947	0.03725	-0.08255	0.06360	-0.05563	1379	-0.2543	0.799
Host2 ✻ Area2	Sheep - Cattle ✻ Jalpaiguri - Alipurduar	0.03229	0.03694	-0.04018	0.10476	0.18961	1379	0.8740	0.382
Season1 ✻ Area1	Summer - Monsoon ✻ Cooch Behar - Alipurduar	-0.03443	0.02405	-0.08161	0.01275	-0.20219	1379	-1.4316	0.152
Season2 ✻ Area1	Winter - Monsoon ✻ Cooch Behar - Alipurduar	-0.01362	0.02569	-0.06403	0.03678	-0.08000	1379	-0.5302	0.596
Season1 ✻ Area2	Summer - Monsoon ✻ Jalpaiguri - Alipurduar	0.02380	0.03573	-0.04630	0.09390	0.13974	1379	0.6660	0.506
Season2 ✻ Area2	Winter - Monsoon ✻ Jalpaiguri - Alipurduar	0.04530	0.03888	-0.03096	0.12156	0.26599	1379	1.1652	0.244

Fixed Effects Parameter Estimates

				95% Confidence Interval					
**Names**	**Effect**	**Estimate**	**SE**	**Lower**	**Upper**	**β**	**df**	**t**	**p**
Host1 ✻ Season1 ✻ Area1	Goat - Cattle ✻ Summer - Monsoon ✻ Cooch Behar - Alipurduar	0.12642	0.05869	0.01129	0.24154	0.74234	1379	2.1541	0.031
Host2 ✻ Season1 ✻ Area1	Sheep - Cattle ✻ Summer - Monsoon ✻ Cooch Behar - Alipurduar	0.05716	0.05687	-0.05440	0.16873	0.33568	1379	1.0052	0.315
Host1 ✻ Season2 ✻ Area1	Goat - Cattle ✻ Winter - Monsoon ✻ Cooch Behar - Alipurduar	0.12593	0.06333	0.00170	0.25016	0.73947	1379	1.9885	0.047
Host2 ✻ Season2 ✻ Area1	Sheep - Cattle ✻ Winter - Monsoon ✻ Cooch Behar - Alipurduar	0.05391	0.06145	-0.06664	0.17446	0.31657	1379	0.8773	0.380
Host1 ✻ Season1 ✻ Area2	Goat - Cattle ✻ Summer - Monsoon ✻ Jalpaiguri - Alipurduar	0.18516	0.08730	0.01391	0.35641	1.08729	1379	2.1211	0.034

Fixed Effects Parameter Estimates

				95% Confidence Interval					
**Names**	**Effect**	**Estimate**	**SE**	**Lower**	**Upper**	**β**	**df**	**t**	**p**
Host2 ✻ Season1 ✻ Area2	Sheep - Cattle ✻ Summer - Monsoon ✻ Jalpaiguri - Alipurduar	0.09171	0.08513	-0.07529	0.25872	0.53855	1379	1.0773	0.282
Host1 ✻ Season2 ✻ Area2	Goat - Cattle ✻ Winter - Monsoon ✻ Jalpaiguri - Alipurduar	0.01142	0.09549	-0.17591	0.19875	0.06705	1379	0.1196	0.905
Host2 ✻ Season2 ✻ Area2	Sheep - Cattle ✻ Winter - Monsoon ✻ Jalpaiguri - Alipurduar	0.00515	0.09367	-0.17859	0.18890	0.03027	1379	0.0550	0.956

Supplementary Table S 10 – Overall infection.

General Linear Model

Model Info

**Info**	
Estimate	Linear model fit by OLS
Call	Overall infection ~ 1 + Host + Season + Area + Host:Season + Host:Area + Season:Area + Host:Season:Area

Model Info

Info	
R-squared	0.0422
Adj. R-squared	0.0242

Model Results

ANOVA Omnibus tests

	SS	df	F	p	η²p	ω²
Model	12.5877	26	2.338	< .001	0.042	0.024
Host	0.0871	2	0.210	0.810	0.000	0.000
Season	4.5392	2	10.962	< .001	0.016	0.014
Area	0.3208	2	0.775	0.461	0.001	0.000
Host ✻ Season	0.7441	4	0.898	0.464	0.003	0.000
Host ✻ Area	0.7490	4	0.904	0.461	0.003	0.000
Season ✻ Area	1.2018	4	1.451	0.215	0.004	0.001
Host ✻ Season ✻ Area	1.0254	8	0.619	0.762	0.004	0.000
Residuals	285.5154	1379				
Total	298.1031	1405				

Fixed Effects Parameter Estimates

				95% Confidence Interval					
Names	Effect	Estimate	SE	Lower	Upper	β	df	t	p
(Intercept)	(Intercept)	0.67932	0.0147	0.6505	0.70818	0.00000	1379	46.17840	< .001
Host1	Goat - Cattle	-0.01911	0.0357	-0.0892	0.05095	-0.04149	1379	-0.53513	0.593
Host2	Sheep - Cattle	0.00256	0.0357	-0.0674	0.07250	0.00555	1379	0.07176	0.943

Fixed Effects Parameter Estimates

Names	Effect	Estimate	SE	95% Confidence Interval Lower	95% Confidence Interval Upper	β	df	t	p
Season1	Summer - Monsoon	-0.06046	0.0345	-0.1281	0.00722	-0.13126	1379	-1.75240	0.080
Season2	Winter - Monsoon	-0.17319	0.0373	-0.2464	-0.09999	-0.37600	1379	-4.64135	< .001
Area1	Cooch Behar - Alipurduar	0.02658	0.0271	-0.0266	0.07978	0.05770	1379	0.98001	0.327
Area2	Jalpaiguri - Alipurduar	-0.01210	0.0408	-0.0922	0.06801	-0.02628	1379	-0.29641	0.767
Host1 ✻ Season1	Goat - Cattle ✻ Summer - Monsoon	-0.08009	0.0837	-0.2443	0.08414	-0.17388	1379	-0.95667	0.339
Host2 ✻ Season1	Sheep - Cattle ✻ Summer - Monsoon	0.05711	0.0827	-0.1051	0.21932	0.12398	1379	0.69064	0.490
Host1 ✻ Season2	Goat - Cattle ✻ Winter - Monsoon	-0.02810	0.0915	-0.2076	0.15138	-0.06100	1379	-0.30709	0.759
Host2 ✻ Season2	Sheep - Cattle ✻ Winter - Monsoon	-0.03465	0.0900	-0.2113	0.14198	-0.07523	1379	-0.38487	0.700
Host1 ✻ Area1	Goat - Cattle ✻ Cooch Behar - Alipurduar	-0.00358	0.0664	-0.1339	0.12672	-0.00777	1379	-0.05389	0.957
Host2 ✻ Area1	Sheep - Cattle ✻ Cooch Behar - Alipurduar	-0.05739	0.0648	-0.1845	0.06970	-0.12460	1379	-0.88584	0.376

Fixed Effects Parameter Estimates

				95% Confidence Interval					
Names	Effect	Estimate	SE	Lower	Upper	β	df	t	p
Host1 ✻ Area2	Goat - Cattle ✻ Jalpaiguri - Alipurduar	0.14026	0.0995	-0.0550	0.33550	0.30450	1379	1.40927	0.159
Host2 ✻ Area2	Sheep - Cattle ✻ Jalpaiguri - Alipurduar	0.06836	0.0987	-0.1253	0.26199	0.14842	1379	0.69261	0.489
Season1 ✻ Area1	Summer - Monsoon ✻ Cooch Behar - Alipurduar	0.04000	0.0643	-0.0861	0.16605	0.08683	1379	0.62245	0.534
Season2 ✻ Area1	Winter - Monsoon ✻ Cooch Behar - Alipurduar	-0.02622	0.0686	-0.1609	0.10844	-0.05693	1379	-0.38200	0.703
Season1 ✻ Area2	Summer - Monsoon ✻ Jalpaiguri - Alipurduar	-0.13941	0.0955	-0.3267	0.04787	-0.30266	1379	-1.46026	0.144
Season2 ✻ Area2	Winter - Monsoon ✻ Jalpaiguri - Alipurduar	-0.01158	0.1039	-0.2153	0.19217	-0.02514	1379	-0.11151	0.911

Fixed Effects Parameter Estimates

Names	Effect	Estimate	SE	95% Confidence Interval Lower	95% Confidence Interval Upper	β	df	t	p
Host1 ✻ Season1 ✻ Area1	Goat - Cattle ✻ Summer - Monsoon ✻ Cooch Behar - Alipurduar	0.08080	0.1568	-0.2268	0.38837	0.17541	1379	0.51532	0.606
Host2 ✻ Season1 ✻ Area1	Sheep - Cattle ✻ Summer - Monsoon ✻ Cooch Behar - Alipurduar	0.15555	0.1519	-0.1425	0.45362	0.33770	1379	1.02377	0.306
Host1 ✻ Season2 ✻ Area1	Goat - Cattle ✻ Winter - Monsoon ✻ Cooch Behar - Alipurduar	-0.14421	0.1692	-0.4761	0.18769	-0.31308	1379	-0.85234	0.394
Host2 ✻ Season2 ✻ Area1	Sheep - Cattle ✻ Winter - Monsoon ✻ Cooch Behar - Alipurduar	0.07180	0.1642	-0.2503	0.39386	0.15587	1379	0.43732	0.662
Host1 ✻ Season1 ✻ Area2	Goat - Cattle ✻ Summer - Monsoon ✻ Jalpaiguri - Alipurduar	-0.22776	0.2332	-0.6853	0.22975	-0.49447	1379	-0.97657	0.329

Fixed Effects Parameter Estimates

				95% Confidence Interval					
**Names**	**Effect**	**Estimate**	**SE**	**Lower**	**Upper**	**β**	**df**	**t**	**p**
Host2 ✻ Season1 ✻ Area2	Sheep - Cattle ✻ Summer - Monsoon ✻ Jalpaiguri - Alipurduar	-0.00170	0.2275	-0.4479	0.44449	-0.00369	1379	-0.00747	0.994
Host1 ✻ Season2 ✻ Area2	Goat - Cattle ✻ Winter - Monsoon ✻ Jalpaiguri - Alipurduar	-0.20393	0.2551	-0.7044	0.29656	-0.44272	1379	-0.79930	0.424
Host2 ✻ Season2 ✻ Area2	Sheep - Cattle ✻ Winter - Monsoon ✻ Jalpaiguri - Alipurduar	0.02400	0.2502	-0.4669	0.51491	0.05211	1379	0.09591	0.924
